# Genomic signatures of admixture and selection are shared among populations of *Zaprionus indianus* across the western hemisphere

**DOI:** 10.1111/mec.16066

**Published:** 2021-07-21

**Authors:** Aaron A. Comeault, Andreas F. Kautt, Daniel R. Matute

**Affiliations:** ^1^ Molecular Ecology and Evolution Group School of Natural Sciences Bangor University Bangor UK; ^2^ Department of Organismic and Evolutionary Biology Harvard University Cambridge Massachusetts USA; ^3^ Department of Biology University of North Carolina Chapel Hill North Carolina USA

**Keywords:** admixture, invasion genetics, local adaptation, range expansion, *Zaprionus indianus*

## Abstract

Introduced species have become an increasingly common component of biological communities around the world. A central goal in invasion biology is therefore to identify the demographic and evolutionary factors that underlie successful introductions. Here we use whole genome sequences, collected from populations in the native and introduced range of the African fig fly, *Zaprionus indianus*, to quantify genetic relationships among them, identify potential sources of the introductions, and test for selection at different spatial scales. We find that geographically widespread populations in the western hemisphere are genetically more similar to each other than to lineages sampled across Africa, and that these populations share a mixture of alleles derived from differentiated African lineages. Using patterns of allele‐sharing and demographic modelling we show that *Z*. *indinaus* have undergone a single expansion across the western hemisphere with admixture between African lineages predating this expansion. We also find support for selection that is shared across populations in the western hemisphere, and in some cases, with a subset of African populations. This suggests either that parallel selection has acted across a large part of *Z*. *indianus's* introduced range; or, more parsimoniously, that *Z*. *indianus* has experienced selection early on during (or prior‐to) its expansion into the western hemisphere. We suggest that the range expansion of *Z*. *indianus* has been facilitated by admixture and selection, and that management of this invasion could focus on minimizing future admixture by controlling the movement of individuals within this region rather than between the western and eastern hemisphere.

## 1 INTRODUCTION

Species that have recently expanded their range and established in historically non‐native regions (i.e., introduced, invasive, or non‐native species) are a nearly ubiquitous feature of contemporary biological communities (Levine, 2008; Simberloff, 2013; Simberloff et al., 2013). Introduced species can have diverse, and frequently negative, impacts on biological communities in their introduced range(s). For example, they may compete with or prey upon native species (Brown et al., 2002; Wanless et al., 2007). Introduced species can also pose economic threats, act as crop pests, and/or vector disease (Pimentel et al., 2005). Understanding the demographic and evolutionary processes that underlie successful biological introductions is therefore an important challenge in modern evolutionary and conservation biology.

Studies of introduced species have identified a number of demographic or evolutionary processes that may contribute to successful introductions (Allendorf & Lundquist, 2003; Whitney & Gabler, 2008). For example, multiple colonization events and hybridization (and subsequent admixture) between differentiated lineages (i.e., populations or species) have both been identified as processes that contribute to genetic diversity within introduced populations (Facon et al., 2008a). Sources of genetic variation within introduced populations have been of broad interest because genetic variation is likely to be important for populations to respond to novel biotic and abiotic selective pressures that they experience in their introduced range. However, multiple colonization events and admixture can also generate novel genotypes that show low fitness when divergent alleles interact negatively, such as when individuals display outbreeding depression (Chapman et al., 2009; Frankham et al., 2011). Processes that increase genetic diversity within introduced populations and the fitness consequences of that genetic diversity need to be understood in order to fully appreciate adaptation (or maladaptation) in introduced species.

Research using phenotypic data has shown that species can rapidly adapt to novel environments they experience in their introduced range (Colautti & Barrett, 2013; Lee, 2002; Prentis et al., 2008). However, it is also possible that adaptations within introduced populations occur before their introductions, conferring traits that help facilitate the successful colonization of new geographic regions. This scenario of adaptation has been termed “anthropogenically induced adaptation to invade” to describe situations where populations adapt to anthropogenic environments (e.g., cities, farmland, or orchards) encountered in their native range prior to range expansion (Hufbauer et al., 2012). Understanding both the shared colonization history and local adaptation within geographically widespread populations of introduced species is therefore central to our understanding of biological introductions and our ability to devise appropriate strategies for their management.

Understanding the geographic origins of introduced populations, alongside the evolutionary processes that operate upon them (e.g., admixture and local adaptation), is also central to our ability to effectively manage biological introductions. For introductions that are geographically widespread, knowledge of shared versus independent aspects of their colonization and evolution will inform whether a single management strategy can be applied across broad geographic regions or whether each introduced population needs to be treated as a unique case. For example, researchers could monitor the movement of individuals between specific sets of countries to limit the opportunity for admixture or the movement of adaptive alleles among regions.

Genetic data are a powerful tool that can be used to estimate demographic and evolutionary events associated with introduced populations or range expansions (Barker et al., 2017; Bock et al., 2015; Dlugosch & Parker, 2008; Fraimout et al., 2017; Kolbe et al., 2004; Lee, 2002; Olazcuaga et al., 2020). Indeed, genetic data have been used to generate insights into biological invasions that include identifying multiple colonization events and admixture within introduced populations (Barker et al., 2017; Dlugosch & Parker, 2008; Facon et al., 2008a; Gibson et al., 2020; Kolbe et al., 2004; Michaelides et al., 2018; Simon et al., 2020). Genome‐scale data can also be used to identify regions of the genome, and candidate genes, with evidence for selection either between native and introduced populations or among populations within species’ introduced ranges (Campbell‐Staton et al., 2020; Olazcuaga et al., 2020). However, demographic analyses of introductions are typically conducted separately, and using independent data, from studies exploring selection (Johri et al., 2020). Whole‐genome sequence data provides a means to jointly explore demographic and selective processes operating within introduced populations, for example, by testing phylogenetic relationships among populations at regions of the genome with evidence of selection compared to putatively neutral regions of the genome. The parallel study of demography and selection in introduced populations has the potential to yield novel insights because it could be used to estimate the geographic or evolutionary origins of alleles that underlie traits that facilitate biological introductions or drive adaptation to novel environments (Calfee et al., 2020). Information on the origins of introductions, from the level of individuals to adaptive alleles, could then be used to inform approaches to mitigate the negative impacts or spread of introductions, in situations where that is the desired outcome (Oduor et al., 2015; Viard et al., 2020).

*Zaprionus indianus* is a generalist fruit fly (Diptera: Drosophilidae; Gupta, 1970) that is thought to be native to sub‐Saharan Africa and can utilize a wide range of fruits as hosts (Yassin & David, 2010). Recently, *Z*. *indianus* has undergone a widespread range expansion into tropical and subtropical regions around the globe, where it is considered a pest species of a wide‐range of fruit crops (Joshi et al., 2014; Leão & Tldon, 2004; van der Linde et al., 2006). *Zaprionus indianus* was first reported in India in 1966 (Gupta, 1970) and, over the subsequent 40 years, has spread east into middle‐eastern Asia and north to Spain (Gibert et al., 2016). More recently, *Z*. *indianus* has expanded its range into the western hemisphere, being reported in São Paulo, Brazil in 1998, with a subsequent, and rapid, expansion across South and Central America. In North America, *Z*. *indianus* was first reported in Florida, USA in 2005 (van der Linde et al., 2006) and by 2011 had spread west to California and north to Pennsylvania, USA (Joshi et al., 2014). Individuals of *Z*. *indianus* have even been collected from stone fruit orchards in southern Ontario and Quebec, Canada (Renkema et al., 2013). Thermal performance curves estimated for lines derived from populations sampled in Florida, North Carolina, and New York in the USA indicate that individuals collected from northern sites have not evolved the ability to tolerate colder temperatures than more southern populations, or populations in their native range (thermal minimum of approximately 12–16℃; Comeault et al., 2020). Current evidence therefore suggests that individuals sampled in northern North America may be migrants that colonize orchards in the relatively warm summer months, and that the range expansion in this region is ongoing. The recent and potentially dynamic nature of *Z*. *indianus's* range expansion into North America highlights the need to better understand genetic relationships among populations and whether those populations have locally adapted to environments in their introduced range. Here, we used whole‐genome sequences collected from native and introduced populations of *Z*. *indianus* to test alternate colonization scenarios, quantify genetic relationships among populations, and explore the geographic (and temporal) dynamics of admixture and selection acting in this species. Our results show that *Z*. *indianus* in the western hemisphere are genetically differentiated from *Z*. *indianus* found across geographically distant locations in Africa and share a pattern of mixed ancestry across their genomes, suggesting an admixture event that predated (or occurred early during) their introduction into the western hemisphere. We also find a strong signal of selection shared across introduced populations in the western hemisphere, consistent with a scenario of either shared selection across introduced populations, or selection in the ancestor of the lineage that went on to colonize regions in the western hemisphere.

## 2 MATERIALS AND METHODS

### 2.1 Population sampling, sequencing, and genotyping

We analysed genome sequences generated from 67 *Z*. *indianus* sampled from four locations in their introduced range (Medellín, Colombia, *n* = 4; Eastern USA, *n* = 25; Hawaii, USA, *n* = 4; India, *n* = 1) and from four locations across their native range in Africa (Zambia: *n* = 6; Kenya: *n* = 7; Senegal: *n* = 14; São Tomé: *n* = 6; Table S1). We also analysed one *Z*. *gabonicus* and 9 *Z*. *africanus* collected from two locations in Africa (São Tome and Kenya). *Zaprionus gabonicus* and *Z*. *africanus* are the two most closely related species to *Z*. *indianus*, and each of these species are reproductively isolated from one another (Yassin et al., 2008). A total of 56 of the genomes in our data set were previously analysed to study levels of genetic diversity across different populations and species of *Zaprionus* (Comeault et al., 2020), and one is from publicly available data used to generate a draft genome assembly for *Z*. *indianus* collected in India (Khanna & Mohanty, 2017). Here we add four genome sequences from a population in South America (Medellín, Colombia) and 14 genomes from five locations in the eastern United States (Florida, Pennsylvania, New Jersey, New York, and North Carolina; see Table S1). All sequences other than the *Z*. *indianus* sample from India were generated from single individuals that were either wild‐caught and preserved in ethanol or collected as a first‐generation offspring of a wild‐caught female. Individuals were sequenced to a mean depth of 20–49× using paired‐end Illumina reads as described in Comeault et al. (2020). The sequence for *Z*. *indianus* from India was generated from two males from a single isofemale line that was collected in Punjab, India (Khanna & Mohanty, 2017).

Raw sequence data was initially parsed and barcodes were removed by the University of North Carolina's high‐throughput sequencing facility. We used the bwa mem algorithm (v0.7.15) to map reads, for each individual, to a previously published *Z*. *indianus* reference genome generated from an isofemale line established from a female collected in Florida in 2014 (Comeault et al., 2020). We sorted and filtered mapped reads using samtools (v1.4), marked duplicates using picard’s *markduplicates* tool (v2.2.4), and realigned around indels using gatk’s realignertargetcreator and indelrealigner tools (v3.8; (McKenna et al., 2010)).

We estimated genotypes for each individual using gatk’s haplotypecaller tool (v3.8) with options “‐‐emitRefConfidence GVCF”, “‐‐minReadsPerAlignmentStart 4”, “‐‐standard_min_confidence_threshold_for_calling 8.0”, and “‐‐minPruning 4” and performed joint genotyping using gatk’s genotypegvcfs tool. We then filtered SNPs using gatk’s VariantFiltration tool with option “‐‐filterExpression “QD < 2.0 || FS > 60.0 || SOR > 3.0 || MQ < 40.0 || MQRankSum < −12.5 || ReadPosRankSum < −8.0””. We hard‐filtered genotypes using vcftools with options “‐‐max‐missing 0.5” and “‐‐mac 2”.

In species with heterogametic sex chromosomes (e.g., *XY* or *ZW* sex determination), the smaller effective population size of the sex chromosomes can exacerbate the impact that genetic bottlenecks have on genetic diversity (Belleghem et al., 2018; Pool & Nielsen, 2007). Lower diversity on the *X* can, in turn, lead to elevated estimates of genetic differentiation along the sex chromosomes (Cruickshank & Hahn, 2014). It is therefore particularly important to consider the genomic location of differentiation in introduced species, as demographic changes (e.g., bottlenecks) are frequently associated with biological introductions (Frankham, 2005; Lee, 2002). To facilitate comparisons between the autosomes and the *X* chromosome, we used coverage to identify putative‐*X* and putative‐autosomal scaffolds in the reference genome used for this study. We calculated normalized mean sequencing depth (i.e., sequencing depth per scaffold divided by mean sequencing depth across all sequenced sites) across scaffolds for five male and four female *Z*. *indianus*, and identified *X* scaffolds as those in which normalized mean sequencing depth was <0.85 for at least four of the five males and >0.85 for at least three of the four females (for details regarding the choice of threshold see Supporting Information and Supporting Information Data; Comeault et al., 2021). Scaffolds smaller than 100 kb were deemed too small to make reliable assignments and scaffolds with a mean normalized read depth >1.5 in at least seven out of the nine samples (independent of sex) were deemed putative repeat elements and not further assigned. Finally, we aimed to identify scaffolds belonging to the *Y*, defined as those with a mean normalized coverage of greater than 0.25 in at least four of the five males and <0.25 in at least three of the four females. This approach did not detect any putatively *Y* scaffolds. Our approach resulted in 94% (137.2 Mb) of the assembly being assigned to autosomes or the X chromosome, with 24.6% (33.7 Mb) being identified as belonging to the *X* chromosome (7.7 Mb were located on scaffolds that were too small and 0.8 Mb on scaffolds with abnormally high coverage). Our estimate of *X* chromosome size is within the range of *X* chromosomes of other Drosophilid flies (e.g., ~23% to 68% of the genome belong to the *X* for *Drosophila melanogaster*, *D*. *busckii*, and *D*. *pseudoobscura*; searched on https://www.ncbi.nlm.nih.gov/genome/).

In addition to the genome‐wide SNP data set, we genotyped each individual's mitochondrial genome. Because mitochondrial haplotypes are maternally inherited and do not undergo recombination, they can be utilized to estimate the number of colonization events occurring during range expansions associated with biological invasions (Facon et al., 2008b; Kolbe et al., 2004; Michaelides et al., 2018). We mapped raw sequence reads, for each individual, to an assembled mitochondria of *Z*. *indianus* (assembled from same isofemale line from Florida used for the reference genome described above; Supporting Information Data) following the same procedure described above for the nuclear genome. Using the processed BAM files, we generated a mitochondrial genome sequence, for each individual, using the ‐doFasta option in angsd (Korneliussen et al., 2014). When calling mitochondrial sequences, we filtered reads with map quality <30 and mapping base quality <18 (‐minMapQ and ‐minQ options, respectively).

### 2.2 Population structure

In 2008 Yassin and colleagues generated mitochondrial haplotype data from *CO*‐*I* and *CO*‐*II* genes sequenced from 23 *Z*. *indianus* populations sampled from Florida, eastern South America, Africa (including Madagascar), and India, but did not find any evidence of population structure associated with geography (Yassin et al., 2008). However, we previously identified relatively strong genetic differentiation among *Z*. *indianus* populations in their native and introduced ranges using whole‐genome data (*F*_ST_ = 0.14–0.19; Comeault et al., 2020). Here we leverage whole‐genome sequence data to further explore relationships among *Z*. *indianus* populations. We first conducted a set of analyses to quantify population structure across our samples. Specifically, we carried out principal component analysis (PCA) and genetic cluster inference with pcangsd (v0.95; Meisner & Albrechtsen, 2018), and phylogenetic analyses using maximum likelihood (ML) and coalescent based approaches with snphylo (Lee et al., 2014) and astral (Rabiee et al., 2019), respectively. pcangsd was run using the ‐*admix* method with default values on genotype likelihoods estimated with angsd using the option “‐SNP_pval 1e‐6” (GATK method; Korneliussen et al., 2014; McKenna et al., 2010). snphylo was run on hard‐filtered SNPs using a linkage disequilibrium (LD) threshold of 0.2 to thin sites and allowing a maximum of 15% of individuals to be missing genotype information at a given site (−l and −M options in snphylo, respectively). astral was run using 7085 gene trees that were randomly sampled from 14,165 gene trees constructed from alignments of phased 500‐SNP nonoverlapping genomic windows generated along the 40 largest scaffolds of our assembly (61.2 Mb or ~42% of the reference genome sequence; Supporting Information). Gene trees were constructed using raxml (v8.2.4; (Stamatakis, 2014)) with optimization of substitution rates under the GTR + GAMMA substitution model and 20 runs on distinct starting trees (see Supporting Information and Supporting Information Data for details).

We also estimated phylogenetic relationships among individuals based on mitochondrial genome sequences. We aligned mitochondrial genomes using the mafft aligner (v7.407) and estimated a mitochondrial tree using raxml (v8.2.4; (Stamatakis, 2014)) with optimization of substitution rates under the GTR + GAMMA substitution model and 20 runs on distinct starting trees. Support was estimated for internal branches of this tree using 100 bootstrap replicates.

Finally, to allow comparisons between autosomes and the *X* chromosome, we calculated nucleotide diversity within nonoverlapping 5 kb genomic windows for each *Z*. *indianus* population using vcftools (v0.1.15; (Danecek et al., 2011)) and *F*_ST_ between populations using angsd (v0.920). When calculating *F*_ST_, we first estimated the site frequency spectrum (SFS) and genotype likelihoods, for each population, using *Z*. *africanus* as the outgroup, and filtered SNPs using options “‐minMapQ 1”, “‐minQ 20”, “‐setMinDepth N*6”, and “‐setMaxDepth N*60” options, where N is the number of individuals sampled from a population. We then estimated all pairwise 2D‐SFS using angsd’s “realSFS” tool, and *F*_ST_ in 5 kb genomic windows using angsd’s “realSFS fst index” and “realSFS fst stats2” tools (Supporting Information Data). We compared median estimates of nucleotide diversity and genetic differentiation within genomic windows on the *X* chromosome and autosomes for populations in *Z*. *indianus's* native range, in their introduced range, and between their introduced and native ranges.

### 2.3 Fine‐scale relationships across the genome

Results from the analyses of population structure described above clearly identify genetic differentiation among *Z*. *indianus* populations at two scales: between populations in the western hemisphere and Africa, and among populations within both native and introduced ranges (see Results). We next explored the relative support for relationships among east African, west African, and introduced (i.e., western hemisphere and India) lineages, across the genome, using “topology weighting by iterative sampling of subtrees” (twisst; Martin & Belleghem, 2017). This approach allowed us to quantify phylogenetic relationships (or shared ancestry) between a focal introduced population (eastern USA, Colombia, Hawaii, or India) and west and east‐African lineages. We estimated topology weights for the three topologies: (i) (*Z*. *africanus* [*Z*. *indianus* east Africa {*Z*. *indianus* west Africa, *Z*. *indianus* “introduced”}]), (ii) (*Z*. *africanus* [*Z*. *indianus* west Africa {*Z*. *indianus* east Africa, *Z*. *indianus* “introduced”}]), and (iii) (*Z*. *africanus* [*Z*. *indianus* “introduced” {*Z*. *indianus* east Africa, *Z*. *indianus* west Africa}]) (see inset of Figure 3a for illustrations). These topologies correspond to phylogenetic relationships where the introduced and west African lineages are more closely related to each other than either are to the east African lineage (hereafter referred to as “west African ancestry”, with respect to the introduced lineage), the introduced and east African lineages are more closely related to each other than either are to the west African lineage (“east African ancestry”), or the two African lineages are more closely related to each other than to the introduced lineage (“diverged”), respectively.

We estimated relative weights for each of the three topologies described above by running twisst on phylogenetic trees estimated for nonoverlapping genomic windows, each containing 500 SNPs, along the 40 largest scaffolds of the reference genome used in this study. Each tree was estimated using maximum likelihood analysis in raxml (v8.2.4; Stamatakis, 2014; see Supporting Information for analysis using pairwise genetic distance and neighbour joining trees). All twisst runs used *Z*. *africanus* as the outgroup, *Z*. *indianus* from São Tomé as the west African lineage, *Z*. *indianus* from Zambia as the east African lineage, and *Z*. *indianus* from either Colombia, east USA, Hawaii, or India as the focal introduced lineage. To speed up run times with twisst, we randomly selected six individuals (12 haplotypes) from the São Tomé population, six individuals from the Zambian population, and six individuals of *Z*. *africanus*, extracted these individual's phased haplotypes from phylip alignments (Supporting Information and Supporting Information Data), and used these individuals when constructing trees. For the different “focal” populations we included all individuals (Hawaii, Colombia, and India) or subsampled seven individuals (Senegal) or 12 individuals (eastern USA). We ran twisst using the “fixed” method with support for the alternate topologies (i.e., topology weights) estimated from a random sample of 600 subtrees per window. We summarized weights as supporting a given topology if more than 50% of sampled subtrees supported that topology. All other windows were classified as “ambiguous”.

Under a scenario where the introduced populations were independently colonized by different populations from the native range, we do not expect patterns of ancestry across the genome to be shared across introduced populations. To test this prediction, we summarized ancestry information for each genomic window, for each introduced population, as the difference between topology weights for topology 1 (shared west African ancestry) and topology 2 (shared east African ancestry). We then calculated correlations in this “ancestry score” between all pairs of focal introduced populations.

In addition to the window‐based approach implemented with twisst, we estimated sharing of derived alleles among populations of *Z*. *indianus* with treemix. Unlike the three‐population window‐based approach we used when running twisst, treemix allows us to test for multiple admixture events and across all populations simultaneously. We first used treemix to infer maximum likelihood ancestry graphs with and without migration among *Z*. *indianus* populations using allele frequency estimates from each population we sampled. We specified *Z*. *africanus* as the outgroup, grouped SNPs in windows of 500 to account for nonindependence among adjacent SNPs (‐k option in treemix), and ran treemix allowing for 0–5 migration events across the tree.

Finally, we tested patterns of allele‐sharing among explicitly defined *Z*. *indianus* populations using four‐population tests as implemented with treemix’s “fourpop” function. This test computes *f*_4_ statistics for trees in the form ([A, B],[C, D]), where a significantly nonzero *f*_4_ value indicates an excess of allele sharing, consistent with introgression, between taxon B and C (negative *f*_4_) or B and D (positive *f*_4_). We computed *f*_4_ statistics holding *Z*. *africanus* as the outgroup (“A” taxon in the aforementioned tree). Because treemix analyses utilize information from allele frequencies within each population, we did not analyse the *Z*. *indianus* sample from India because this sequence was derived from two males collected from a single isofemale line.

### 2.4 Demographic modelling

We took a demographic modelling approach to explore support for different demographic events occurring during *Z*. *indianus's* expansion into the western hemisphere. We first computed SFS for populations sampled in São Tomé, Zambia, and the eastern USA using angsd (v0.920) with options “‐domajorminor 1 ‐gl 1 ‐domaf 1 ‐dogeno 3 ‐doCounts 1 ‐dopost 2 ‐doHWE 1 ‐minHWEpval 0.01 ‐minMapQ 20 ‐minQ 20 ‐doSaf 1”. The two African populations were chosen to reflect the two differentiated lineages we sampled in African and the population from the eastern USA was chosen because we had the largest sample size from this population (24 individuals) and this population represents the most recently colonized in the western hemisphere. SFS were polarized using *Z*. *africanus* (‐anc option in angsd). We then estimated the joint‐SFS for the three focal populations using angsd’s “realSFS” function.

We inferred demographic parameters and model‐fit under 8 different demographic scenarios using the unfolded joint‐SFS and fastsimcoal2 v.2.6.0.3 (Excoffier et al., 2013). Three demographic scenarios allowed for a split between the eastern USA population (i.e., the North American lineage) and one of the African lineages (i.e., the Zambian lineage, the São Toméan lineage, or the ancestral African lineage; histories C, D and A; Figures S1–S3). A fourth scenario modelled the North American lineage being simultaneously colonized by both African lineages, with the African lineages having differentiated with gene flow before founding the North American lineage (history B; Figures S1–S3). The remaining four demographic scenarios allowed for a combination of differentiation and admixture between lineages, with admixture either occurring before the North American lineage split from an African lineage, or after this split (histories E–H; Figures S1–S3). We chose these eight scenarios because they allowed us to quantify support for or against a history containing admixture (compare histories A–D vs. histories E–H; Figures S1–S3) and whether admixture was most likely to occur before or after the North American lineage split from an African ancestor (compare histories E & F to histories G & H; Figures S1–S3). Finally, we fit each demographic scenario three times, either with no within‐lineage change in population size (Figure S1), with exponential growth in the three contemporary lineages (Figure S2), or with exponential growth in the North American lineage and discrete changes in population size for both African lineages and the North American lineage (Figure S3). Each model was independently fit to the joint‐SFS in 100 independent fastsimcoal runs, each optimizing parameters for 100 ECM cycles and estimating the expected SFS using 200,000 coalescent simulations (Supporting Information for details). We report AIC and inferred demographic parameter maximum‐likelihood point estimates for the three runs that received the lowest AIC under each demographic scenario.

### 2.5 Local and shared selection experienced during range expansion

Results from the analyses of genetic differentiation introduced above support a scenario where *Z*. *indianus* colonized the Americas from the same ancestral population and/or with a shared out‐of‐Africa demographic history. However, they do not preclude the possibility that different introduced populations are adapting to different local environments using different adaptive genetic variation. We used two approaches to test for shared versus independent selection acting across introduced populations of *Z*. *indianus* in the Americas (i.e., those from Colombia, Hawaii, and eastern USA).

First, we estimated selection within individual populations using the population branch statistic (PBS; Yi et al., 2010). We estimated the PBS at three levels of comparison: (i) each introduced population was compared to the Zambia and São Tomé populations (“between‐range” comparisons), (ii) each introduced population was compared to the other two introduced populations from the western hemisphere (“within‐introduced range” comparisons), and (iii) the Zambia and São Tomé populations were each compared to the two African populations they were most differentiated from (i.e., west African populations [São Tomé and Senegal] or east African populations [Zambia and Kenya], respectively; “within‐native range” comparisons). These three levels of comparison allowed us to identify loci that show accentuated differentiation in a given introduced population relative to west‐ or east‐African lineages, among introduced populations, and among native populations, respectively. We estimated PBS within nonoverlapping 5 kb windows using angsd (v0.920) as described above when estimating *F*_ST_ (section 2.2). Because this approach was based on windows, we analysed windows on scaffolds identified as putatively belonging to the X chromosome separately from those found along putatively autosomal scaffolds. Genomic windows with a PBS in the top 99% of values for windows found on X scaffolds or along autosomal scaffolds were classified as outliers. Windows that were outliers in more than one of the introduced populations (when compared against west and east African populations) were classified as “shared invasive outlier” windows. These windows have the strongest evidence of experiencing selection early during *Z*. *indianus's* expansion into their introduced range or being subject to parallel selection in multiple introduced populations. Genomic windows that experience selection within a single introduced population were identified based on having PBS in the top 99% of values in the analysis that included all three introduced populations (i.e., “within‐introduced range” comparison). Finally, genomic windows with a PBS in the top 99% of values in a within‐native range comparison were classified as windows experiencing selection in *Z*. *indianus's* native range. We tested whether the overlap in the number of outlier windows identified among these different comparisons was greater than expected by chance using randomization tests.

We also used baypass (v2.2; Gautier, 2015) and an independent test of evidence of shared selection across all introduced populations in the western hemisphere. baypass implements a Bayesian hierarchical model (Coop et al., 2010; Gautier, 2015) to estimate loci that show accentuated differentiation among populations and allows the user to specify covariables to test for associations between loci and those covariables. For this analysis, we used geography as a covariable (western hemisphere vs. Africa) and identified SNPs that showed allele frequency differences between the western hemisphere and Africa with the *C*_2_ contrast statistic (Olazcuaga et al., 2020). We considered SNPs with an empirical *p*‐value < .05 and FDR < 0.1 as the best candidates for being subject to selection between *Z*. *indianus's* introduced and native ranges (see Supporting Information for further details).

## 3 RESULTS

### 3.1 Genotyping

Using whole‐genome sequence data we identified 22,911,754 hard‐filtered SNPs across all samples included in this study. As expected, when comparing across species, the majority of sites were fixed between *Z*. *africanus* and *Z*. *indianus* samples and fewer SNPs segregated within populations of *Z*. *indianus*. Within *Z*. *indianus*, the number of segregating sites was lower in populations sampled in the introduced range (~2.9 to 5.7 million SNPs; Hawaiian and eastern USA populations, respectively) compared to the native range (~6.8 to 10.7 million SNPs; Zambian and Senegalese populations, respectively).

### 3.2 Population structure

**FIGURE 1:**
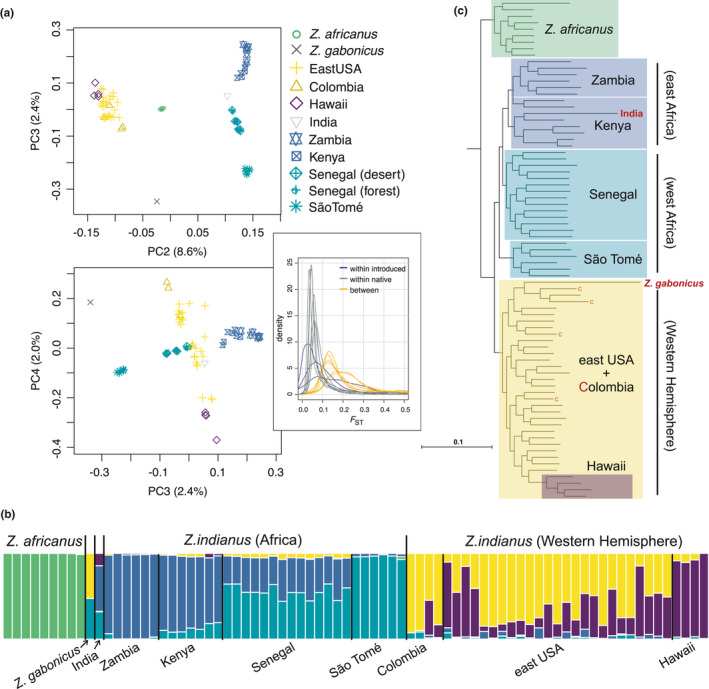
Population structure across native and invasive populations of *Zaprionus indianus*. (a) Principal component analysis carried out using pcangsd identifies genetic differentiation between native and invasive populations (PC2) and, to a lesser extent, among native (PC3) and invasive populations (PC4). Inset in panel (a) shows pairwise *F*_ST_ for these three levels of comparison calculated across 5 kb genomic windows. (b) Genetic clustering analysis supports four genetic clusters within *Z*. *indianus*, reflecting differentiation among eastern and western Africa in the native range and Colombia and Hawaii in the invasive range. (c) Phylogenetic tree estimated using snphylo supports differentiation between east African, west African, and North American populations of *Z*. *indianus*. Tips of the tree representing samples from Colombia are indicated with a red “C”. See Figure S15 for details showing no substructure within sample locations

Principal component analysis (PCA), genetic clustering, and phylogenetic analyses all identified genetic differentiation between introduced and native populations (Figure 1). Among *Z*. *indianus* samples, the majority of genetic variation was structured between individuals from introduced and native parts of the species’ range (PC2 accounts for ~8.6% of genetic variation; Figure 1a; PC1 separates the two species *Z*. *indianus* and *Z*. *africanus* and accounts for 39.0% of genetic variation [not shown]). Phylogenetic analysis with snphylo and astral provided further support for differentiation between introduced and native populations: all individuals sampled from the western hemisphere were more closely related to each other than to individuals sampled from Africa, and all African individuals were more closely related to each other than to individuals from the western Hemisphere (Figure 1c; Figure S4 for astral tree). Phylogenetic analyses also indicate differentiation between west African and east African populations (Figure 1c; Figure S4 for astral tree).

*F*_ST_ estimated within 5kb genomic windows was highest between native and introduced populations (median [pairwise] *F*_ST_: range = 0.154–0.230; orange curves in Figure 1a inset; Table S2). The strongest genetic differentiation between native and introduced populations was observed between the Hawaiian and Zambian populations (median *F*_ST_ = 0.230) and the Hawaiian and São Toméan populations (median *F*_ST_ = 0.220). The strongest genetic differentiation within the native range was between Kenyan and São Toméan populations (median *F*_ST_ = 0.084) followed by Zambian and São Toméan populations (median *F*_ST_ = 0.077). Among introduced populations, populations from Colombia and Hawaii were the most differentiated (median *F*_ST_ = 0.160), with the eastern USA samples being less differentiated from both Colombia (median *F*_ST_ = 0.042) and Hawaii (median *F*_ST_ = 0.089). Consistent with patterns of genetic differentiation, clustering analysis grouped *Z*. *indianius* into four genetic clusters loosely defining individuals with ancestry from west Africa, east Africa, Colombia, or Hawaii (Figure 1b).

**FIGURE 2:**
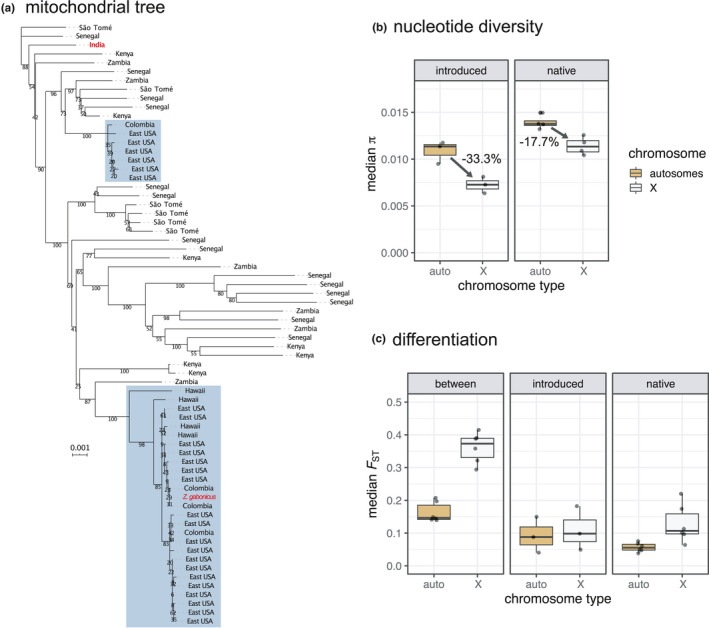
Differentiation within different genomic regions. (a) Phylogenetic relationships among mitochondrial haplotypes sampled from populations in *Zaprionus indianus's* introduced and native ranges. Individuals from the western hemisphere are highlighted with blue boxes while the sample from India and *Z*. *gabonicus* are indicated with red text. (b) The percent reduction in median nucleotide diversity (*π*) on the *X* chromosome relative to the autosomes tends to be larger in populations of *Z*. *indianus* in their introduced compared to their native range (mean % differences shown). (c) Differentiation (*F*_ST_) is highest along the *X* chromosome in pairwise comparisons between populations in the introduced and native ranges (“between” panel) and is also higher on the *X* chromosome compared to the autosomes in pairwise comparisons between populations in the native range (“native” panel)

Median genetic differentiation was particularly high within genomic windows on scaffolds that putatively make up the *X* chromosome, being 2–2.7 times higher than genetic differentiation along the autosomes in comparisons between populations in the western Hemisphere and Africa, 1.1–1.2 times higher in comparisons between populations within the western Hemisphere, and 1.3–3.3 times higher in comparisons between populations within Africa (Figure 2c). Median nucleotide diversity (π) also differed between the *X* chromosome and the autosomes, and was 28.6%–38.1% lower on the *X* chromosome relative to the autosomes in introduced populations and 8.5%–30.3% lower on the *X* relative to the autosomes in native populations (Figure 2b).

**FIGURE 3:**
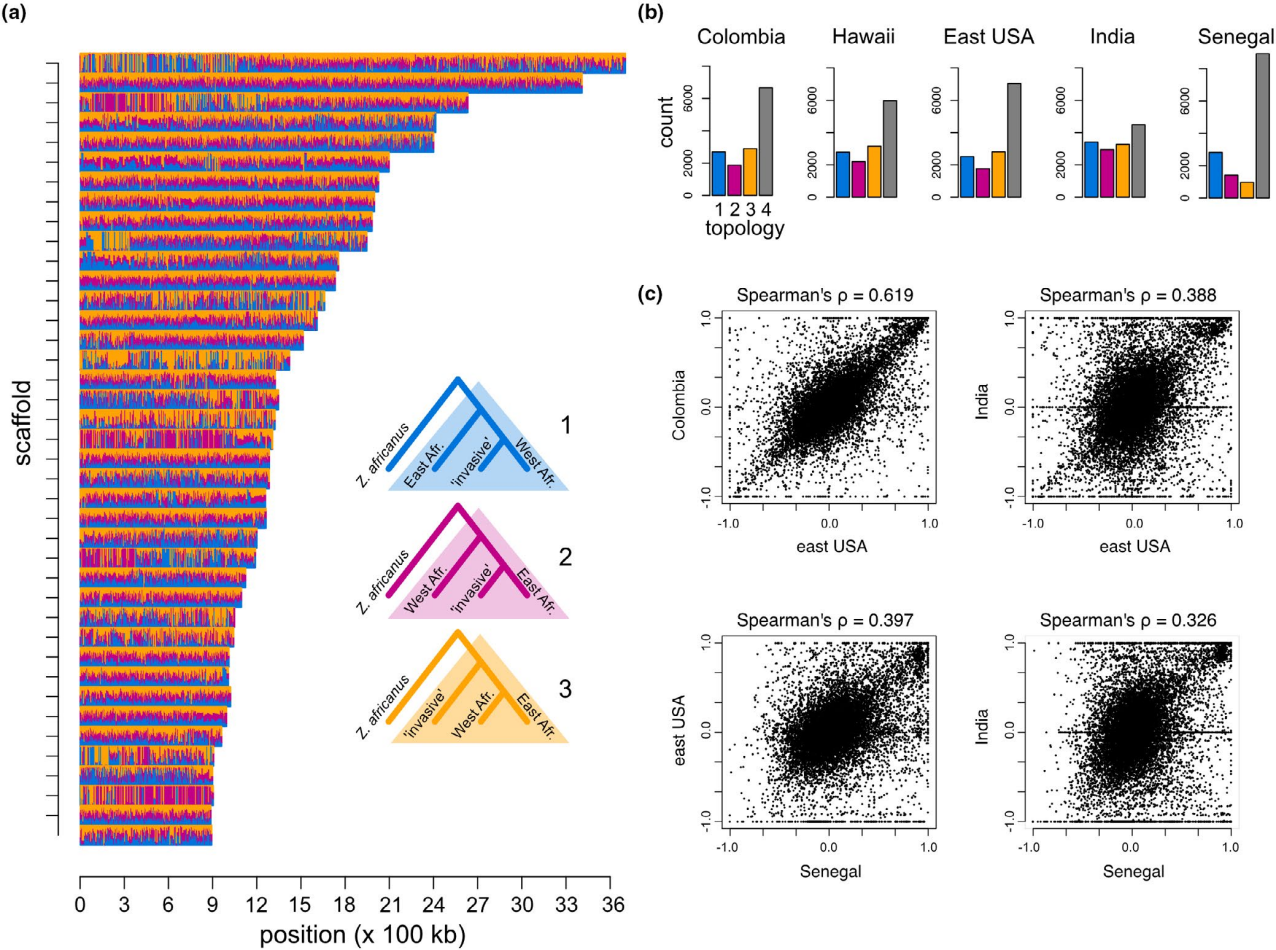
Widespread variation in phylogenetic relationships across the genomes of *Zaprionus indianus*. Vertical bars in (a) represent genomic windows and are coloured based on weights for each of the three possible four‐taxon unrooted topologies (see inset). We find that, for the majority of windows, support for any one of the three topologies tended to be ambiguous (i.e., all topologies received support less than 0.5). (b) Counts of windows that supported different topologies, for each population tested. Blue bars show the number of windows that support shared west‐African ancestry (topology 1), pink bars shared east‐African ancestry (topology 2), orange bars “divergent” ancestry (topology 3), and grey bars where neither of the three tested topologies received a majority of topology weight (“topology” 4). (c) The difference in support between west‐African and east‐African ancestry (proportion of twisst iterations) was highly correlated across genomic windows between populations sampled in the western hemisphere (c; top left panel) and less correlated between populations in the western hemisphere and India (c; top right panel) or Africa (c; bottom left panel), or between India and Africa (c; bottom right panel)

Levels of genetic differentiation provide evidence of moderately strong differentiation between *Z*. *indianus* from the western hemisphere and Africa, with accentuated differentiation along the *X* chromosome relative to the autosomes. Results also suggest a shared range expansion into the western hemisphere rather than independent colonization from the same or different location in Africa. The single *Z*. *indianus* sample from India was consistently more closely related to individuals from Africa than to any of the individuals sampled in the western hemisphere (Figure 1; however, see astral tree in Figure S4, where the Indian sample is between populations sampled from African and the western hemisphere). This suggests a separate range expansion in the eastern hemisphere or ongoing gene flow between African and Indian populations. The former scenario has also been suggested in a previous analysis of two mitochondrial markers (Yassin et al., 2008).

Phylogenetic analysis of mitochondrial genomes showed no clear structure of mitochondrial (mt) haplotypes across African populations and two distinct mitochondrial lineages present within the western hemisphere (Figure 2a). The majority of *Z*. *indianus* sampled in the eastern USA carried mt haplotypes from the subclade of haplotypes that included all four Hawaiian haplotypes. However, six individuals from the eastern USA possessed mt haplotypes more closely related to a haplotype carried by one of the individuals we sampled in Medellín, Colombia. Patterns of haplotype structure therefore suggest that the western hemisphere was colonized by at least two divergent mt haplotypes and the eastern USA was colonized by individuals carrying both South American and Hawaiian mt haplotypes. This analysis also suggests a relatively recent expansion within Africa, with no notable phylogenetic clustering of mt haplotypes among individuals sampled from this continent (Figure 2a). The single *Z*. *gabonicus* carries a mt haplotype similar to the *Z*. *indianus* from the western hemisphere. This individual also clusters with *Z*. *indianus* from the western hemisphere in analyses using genome‐wide data (Figure 1a,b). However, we do not know the exact collection location or history of the isofemale line that this individual is from, so do not analyse it further.

### 3.3 Fine‐scale relationships across the genome

We next took a phylogenomic approach to test relationships among populations across nonoverlapping genomic windows, each containing 500 SNPs. Topology weights provide evidence for ongoing lineage sorting and weak phylogenetic divergence across *Z*. *indianus* populations (Figure 3). Specifically, the most common outcome of the TWISST analysis was that a window received ambiguous support (i.e., all three topologies shown in Figure 3a received a weight <0.5; range across comparisons: 31.36%–62.74% of windows; grey bars in Figure 3b). Ambiguous support could be the result of ongoing lineage sorting (i.e., a lack of differentiation or divergence) or a lack of sufficient variation to construct a reliable phylogenetic tree. We found that windows that received ambiguous support had, on average, 83.5 segregating sites, while windows that received support for one of the alternate topologies had an average of 87.9–93.2 segregating sites (Figure S5). However, many windows classified as “ambiguous” contained a number of segregating sites similar to windows that received unambiguous topology weights, suggesting that ambiguous support was driven by a lack of differentiation rather than a lack of information (Figure S5).

For populations in the western hemisphere, 20.07%–22.44% of the genome was classified as being diverged from both African lineages (orange topology in Figure 3a; orange bars in Figure 3b), 17.98%–19.87% shared ancestry with the west‐African lineage (blue topology in Figure 3a; blue bars in Figure 3b), and 12.69%–15.82% shared ancestry with the east‐African lineage (pink topology in Figure 3a; pink bars in Figure 3b). Topology weights therefore indicate that populations of *Z*. *indianus* in the western hemisphere have diverged from African lineages, but also that they retain a significant proportion of both west and east African ancestry across their genomes.

Compared to populations in the western hemisphere, the sample from India had fewer windows with ambiguous support (31.36%; Figure 3b) and more support for mixed ancestry across the genome (Figure 3b). By contrast, the population from Senegal showed the highest proportion of the genome receiving ambiguous weights (62.74%; Figure 3b), followed by support for shared west‐African (20.11%; Figure 3b) and east‐African (10.12%; Figure 3b) ancestry. Populations sampled from across *Z*. *indianus's* native and introduced ranges therefore display mixed or mosaic ancestry across the genome and, in some cases, localized divergence (orange topology in Figure 3).

Topology weights were highly correlated across the genome for introduced populations in the western hemisphere (Spearman's ⍴: .565 to .619; all *p* < .001; top left panel in Figure 3c). By contrast, this correlation was much weaker between introduced populations in the western hemisphere and India (⍴: .388 to .405; all *p* < .001; top right panel in Figure 3c) or Senegal (⍴: .360 to .397; all *p* < .001; bottom left panel in Figure 3c), and between the populations in India and Senegal (⍴ = .326; *p* < .001; bottom right panel in Figure 3c). The stronger correlations in topology weights between introduced populations in the western hemisphere compared to between populations in the western hemisphere and India suggest independent introductions to these two regions. Populations in the western hemisphere, however, share a signal of a single admixture event across their genomes, or selection that is widespread across the genome and constrains ancestry at different genomic regions. We conducted additional analyses to test the hypothesis that populations in the western hemisphere share a genomic signature of a single admixture event that predated a shared range expansion across the western hemisphere (Section 3.4).

**FIGURE 4:**
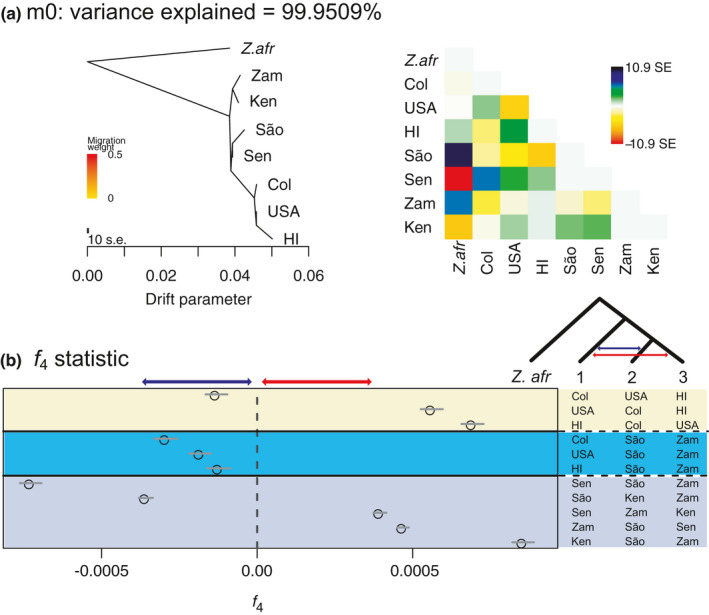
Allele sharing among *Zaprionus indianus* populations. (a) Analyses with treemix identify shared drift among populations within west Africa, east Africa, and the western hemisphere. The matrix in panel (a) shows the fit of residuals to the model (standard error; SE). Darker red and blue/black values represent population pairs where patterns of allele sharing are not well modeled. (b) Allele sharing, as summarized by the *f*_4_ statistic, supports the phylogeny presented in (a) but also provides evidence for admixture among populations in the western hemisphere (comparisons in yellow box), between the African populations (São Tomé and Zambia) and populations in the western hemisphere (comparisons in teal box), and among populations in Africa (comparisons in purple box). Note that not all combinations of populations are shown

We analysed allele frequencies to estimate support for admixture and/or allele sharing among populations of *Z*. *indianus* in their native and introduced ranges. Analyses with treemix supported shared ancestry among the three introduced populations in the western hemisphere and differentiation from African populations (Figure 4a). While increasing the number of migration edges inferred by treemix increased the percent variance explained by the model (and the log likelihood of the model), the model with no migration already accounts for 99.95% of covariance in allele frequencies among populations (Figure S6). In models that allowed for migration, the most consistent evidence for migration was found between populations in Zambia and Kenya and Colombia and Senegal (Figure S6). However, modeling shared drift among populations does not unambiguously support admixture events among populations of *Z*. *indianus*.

In contrast to models of shared drift, analyses of allele sharing among trios of populations (as summarized by the *f*_4_ statistic) provide support for admixture among certain populations. The strongest evidence for admixture was among populations sampled in *Z*. *indianus's* native range (*f*_4_ = −7.28 × 10^−4^ to 8.55 × 10^−4^; *Z* = −41.54 to 50.01; comparisons highlighted in purple in Figure 4b). Admixture was also evident among populations in the western hemisphere, with individuals from the eastern USA being most closely related to individuals from Hawaii, but also being enriched for alleles shared with individuals from Colombia (*f*_4_ = −1.30 × 10^−4^; *Z* = −7.46). Evidence for admixture from the *f*_4_ statistic and twisst (Figures 4b and 3, respectively) therefore both support admixture among populations in Africa and mixed African ancestry within introduced populations in the western hemisphere.

### 3.4 Demographic modelling

We conducted demographic modelling to test whether our data supported a demographic history including admixture over histories that lacked admixture (Figures S1–S3). Across eight different demographic histories, each run under three different scenarios of population size change, those that modeled an admixture event were consistently preferred over those without admixture (Table S3). Across the three scenarios of population size change, the one that allowed for exponential growth in each contemporary lineage was strongly preferred over scenarios where populations were either a constant size or experienced discrete changes in size. The overall best‐fit demographic history was one where the North American lineage was colonized by the western African lineage after there was a large admixture event between the two African lineages (scenario F; Figure S2). A demographic scenario with admixture between the African lineages prior to the North American lineage splitting from the African lineages, and exponential growth in each population, was supported over scenarios where there was no admixture, or where admixture occurred after the North American lineage split from one of the African lineages (Table S3). Under the best‐fit demographic scenario, the timing of the split between the North American and African lineages was 603 generations ago (~35–60 years ago assuming a generation time between 3 and 5 weeks) and the timing of admixture between the two African lineages was 427,950 generations ago, with the African lineages experiencing ongoing gene flow throughout their history (Table S4).

### 3.5 Selection among introduced populations of *Z*. *indianus*

**FIGURE 5:**
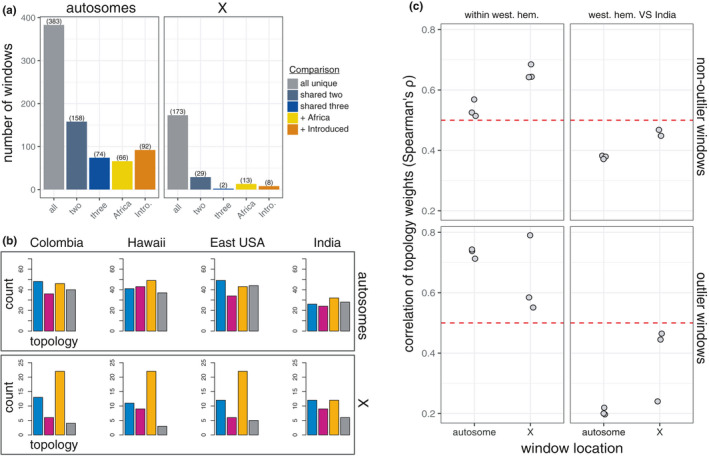
Evidence of shared selection among populations of *Zaprionus indianus* in the western hemisphere. (a) Overlap in outlier windows in different population comparisons. Grey bars show the number of unique windows with evidence of selection (“all unique”) based on the PBS being in the top 99% for at least one of the comparisons between a population in the western hemisphere and African populations. Blue‐grey and blue bars show the number of windows with evidence of selection in two (“shared two”) or all three (“shared three”) of the comparisons made between populations in the western hemisphere and those in Africa. Yellow and orange bars show the number of “all unique” windows that also display evidence of selection within Africa (“+Africa”) or among introduced populations (“+Introduced”). (b) Ancestry estimates based on topology weights reported from twisst for genomic windows that overlapped a window with evidence of selection in at least two introduced populations in the western hemisphere (“shared two” category in panel (a); refer to Figure 3 for summaries of genome‐wide patterns). (c) Correlation in ancestry estimates from twisst between populations in the western hemisphere (“within west. hem.”) versus between populations in the western hemisphere and the sample from India (“west. hem. vs. India”) for different genomic regions

We next estimated regions of the genome with evidence of selection by comparing allele frequency differences (i) between introduced populations in the western hemisphere and native populations in São Tomé and Zambia, (ii) among introduced populations in the western hemisphere, and (iii) across both introduced and native populations. Analyses using the PBS classified 1047 and 389 unique 5 kb genomic windows (~5.1% and ~5.8% of windows; autosomal vs. X chromosome windows, respectively) as outliers based on having a PBS value in the top 99% of windows in comparisons made between introduced and native populations (between‐range comparisons), among introduced populations (within‐introduced range comparisons), and among native populations (within‐native range comparisons). To determine the degree to which these outlier windows were shared among different populations we focused on the 383 and 173 unique windows identified on the autosomes and *X* chromosome, respectively, in between‐range comparisons, as these windows represent those with evidence of being subject to selection in the introduced part of the species’ range. 158 (41.3%) and 29 (16.8%) unique outlier windows identified in between‐range comparisons were identified as outliers in at least two of the three populations from the western hemisphere (blue‐grey bars; Figure 5a; Manhattan plots of *F*_ST_ along scaffolds containing at least one of these windows are given in Figure S7–S13), and 74 (19.3%) and 2 (1.2%) were identified in all three populations (blue bars; Figure 5a), for windows on the autosomes and *X* chromosome, respectively. We generated expected numbers of overlapping outlier windows by randomization (100,000 replicates) and found that for all comparisons made above, the observed number of overlapping windows was greater than expected by chance (all empirical *p*‐values < .0001; Figure S14). Only the situation where 2 windows were shared among all three populations was recovered during randomization, and this was only in 1 of the 100,000 independent samples.

We also identified 66 (autosomal) and 13 (*X* chromosome) windows that were outliers in at least one of the three between‐range comparisons and at least one of the two within‐native range comparisons (17.2% and 7.5% of unique outlier windows; orange bars in Figure 5a; empirical *p*‐value from randomization = 0; Figure S14). These windows represent a shared signal of differentiation between populations in the western hemisphere and Africa, and among African populations. Finally, we found 92 (autosomal) and 8 (*X* chromosome) windows that were outliers in between‐range and among‐introduced comparisons (24.0% and 4.6% of unique outlier windows; gold bars in Figure 5a; empirical *p*‐value from randomization = 0 and 0.034, respectively; Figure S14). These windows represent regions of the genome that are differentiated between populations in the western hemisphere and Africa, but also show accentuated differentiation in at least one population in the western hemisphere relative to the others.

Analysis with baypass identified 98 SNPs as outliers, all of which were located in outlier windows identified in the PBS analysis. baypass outliers were distributed across 19 scaffolds. Nine of these SNPs are located on putatively autosomal scaffolds and 89 are on putatively *X* scaffolds.

We used topology weights inferred with twisst to estimate the origin of putatively adaptive alleles with evidence of selection in at least two of the between‐range comparisons (i.e., 158 and 29 PBS outlier windows on the autosomes and *X* chromosome, respectively; blue‐grey bars in Figure 5a). We focused on these windows because they represent the windows with the strongest evidence of being under selection in introduced populations in the western hemisphere, while still providing a reasonable number of windows to explore ancestry across. We found that the distribution of ancestry (i.e., topology weights) across these outlier windows was significantly different from the genomic background for all three populations in the introduced range in the western hemisphere (Pearson's Chi‐squared tests; all *p* < .03; Figure 5b). Most notably, putatively selected genomic windows were much more likely to support a topology where the introduced population has diverged from both African lineages and less likely to be in a genomic region with ambiguous phylogenetic relationships (Figures 3 and 5b; orange and grey bars, respectively). This pattern is not surprising given that the PBS relies on differentiation to identify putatively selected regions. We also found a stronger correlation in ancestry across autosomal outlier windows compared to nonoutlier windows in all pairwise comparisons between populations from the western hemisphere (Figure 5c), but this was only the case for one out of the three pairwise comparisons for outliers found along the *X* chromosome (Figure 5c). Ancestry estimates across regions of the genome with evidence for selection therefore provide additional support for a signal of directional selection that is shared across introduced populations in the western hemisphere, and also suggests that genomic regions carrying east or west‐African ancestry have both been subject to selection in *Z*. *indianus's* introduced range in the western hemisphere.

## 4 DISCUSSION

Introduced species are becoming an ubiquitous feature of biological communities around the globe (Capinha et al., 2015; Helmus et al., 2014; Levine, 2008; Simberloff et al., 2013). As such, understanding the demographic and evolutionary origins of introduced species is important if we are to understand evolution and biodiversity in the Anthropocene. Research has shown how introduced populations can rapidly differentiate from populations in their native range and adapt to their introduced range (Dupuis et al., 2018; Koch et al., 2020; Ma et al., 2020; Stuart et al., 2021; Zayed & Whitfield, 2008); however, the origin of that adaptive variation is seldom known (however, see Calfee et al., 2020). In this manuscript, we leveraged genome sequences to provide evidence that introduced populations of *Z*. *indianus* found at geographically distant locations in the western hemisphere share ancestry with differentiated populations across their native African range (Figures 3 and 4), have a shared set of loci that have been subject to selection in the western hemisphere (Figure 5a), and that those loci are located in genomic regions carrying west‐African and east‐African ancestry (Figure 5b). Below we discuss how patterns of variation shared among the genomes of these flies helps inform our understanding of the evolutionary processes shaping their introduction into the western hemisphere.

### 4.1 Differentiation among populations

We found evidence for genome‐wide genetic differentiation between geographically‐distant populations in the western hemisphere and native populations in Africa (Figures 1, 2, 3, 4). Using phylogenomic analysis, we show that the introduced populations in the western hemisphere also harbour ancestry shared with both east and west‐African lineages, and that ancestry estimates are correlated across the genomes of individuals from populations in the western hemisphere (Figure 3). Introduced populations in South America and North America therefore carry a genomic signature of a shared range expansion, and demographic modelling supports a scenario in which admixture between differentiated populations in the native range predated *Z*. *indianus's* range expansion into the western hemisphere. Given the dates for when *Z*. *indianus* began to be reported within different regions of South and North America, a likely colonization scenario is one where *Z*. *indianus* colonized eastern South America in the late 1990s (van der Linde et al., 2006) and has, from there, expanded their range eastward and northward, now being found as far north as southern Canada (Joshi et al., 2014; Renkema et al., 2013). Alternatively, it is possible that a population of *Z*. *indianus* that is not included in our sample, and that has diverged from African populations, has independently colonized different locations in the western hemisphere. Further geographic sampling is required to test this hypothesis.

Patterns of genetic differentiation among populations varied across the genome. For example, differentiation between populations in the western hemisphere and those in Africa, and among populations within Africa, was approximately twice as high on the *X* chromosome compared to the autosomes (Figure 2c). In diploid sexual species with heterogametic sex chromosomes, sex chromosomes have a smaller effective population size than the autosomes. This can result in the *X* chromosome (or *Z* chromosome in species with *ZW* sex determination) losing a larger amount of genetic diversity than autosomes during demographic bottlenecks (Charlesworth, 2009; Ellegren, 2009). Interestingly, our results suggests that populations of *Z*. *indianus* in both their introduced and native ranges have experienced recent demographic events, such as bottlenecks. Our phylogenetic analysis of mitochondrial haplotypes also (Figure 2a) support a scenario of a recent range expansion within Africa, as we observed no geographic structure in mitochondrial haplotypes, despite genetic differentiation across *X*‐linked genomic windows (Figure 2a,c, respectively). Similar patterns of differentiation across the autosomes and *X* chromosomes have been reported in introduced populations of *Drosophila suzukii*, both among introduced populations (Koch et al., 2020) and between introduced and native populations (Olazcuaga et al., 2020). Comparing patterns of differentiation among different genomic regions (i.e., the autosomes, *X* chromosome, and mitochondrial genome) highlight the usefulness of genome‐scale data when testing demographic histories of introduced species in both their introduced and native ranges.

### 4.2 A signal of admixture in introduced populations of *Z*. *indianus*

Admixture has been shown in a number of species that have recently expanded their ranges in association with human activities. Introduced populations of the Iguanian lizard *Anolis sagrei* have signatures of mixed ancestry consistent with colonization from multiple regions in their ancestral range (Kolbe et al., 2004). Kolbe et al. (2004) even suggest that *A*. *sagrei's* expansion in their introduced range only proceeded after genetic diversity derived from independent introductions built up within early colonizing populations in the introduced range. Recent work in other systems—introduced wild tomato (*Solanum pimpinellifolium*) on the Galapagos islands and introduced populations of *Mytilus* mussels—provide examples where admixture, or introgression, occurs either during or after species are introduced into novel environments (Gibson et al., 2020; Popovic et al., 2020; Simon et al., 2020). The dynamics of admixture in these systems, and those that we report here for *Z*. *indianus*, highlight how the timing of admixture can vary among taxa and can occur (i) during colonization and range expansion (*A*. *sagrei and Mytilus* spp.), (ii) after being introduced into a novel geographic region (*S*. *pimpinellifolium* and *Mytilus* spp.), or (iii) prior to introduction into a novel geographic region (*Z*. *indianus*). Temporal dynamics such as these are important to consider both from the perspectives of better understanding the evolution of introduced species and in devising appropriate strategies for their management. For example, management strategies could use information on the temporal and geographic dynamics of admixture to prioritize blocking the movement of individuals either between their introduced and native ranges or within their introduced and native ranges. More generally, knowledge of introduced species’ colonization history and adaptation can be used to mitigate the negative impacts that introduced species can have on ecosystems (Oduor et al., 2015; Viard et al., 2020). An important aspect of admixture in introduced species that requires further study is the phenotypic effects of that admixture and whether admixture actively promotes or facilitates range expansion and/or adaptation to novel environments.

### 4.3 Selection in the introduced range

Similar to admixture, populations of introduced species can experience selection at different times and locations during their range expansions and there is increasing evidence of rapid genetic differentiation consistent with selection acting within populations of introduced species (Dupuis et al., 2018; Koch et al., 2020; Ma et al., 2020; Stuart et al., 2021; Zayed & Whitfield, 2008). We found that populations of *Z*. *indianus* in the western hemisphere carry evidence of shared selection based on regions of accentuated differentiation from African populations (Figure 5a). For example, we identified genomic regions with a high PBS across multiple introduced populations or, in some cases, across all three populations (Figure 5a). We also identified windows that were differentiated between introduced populations and African populations, among African populations, and among populations within the introduced range. The latter two scenarios suggest that regions of the genome have experienced selection both across the introduced and native regions, and within individual introduced populations in different parts of their introduced range, respectively. Together with the signature of admixture carried by the introduced populations, these results indicate that the expansion of *Z*. *indianus* populations into the western Hemisphere may have been facilitated by both admixture and selection that occurred early on during (or preceding) this range expansion.

Genomic windows with evidence of selection in at least one of the comparisons we made overlap a total of 658 unique genes that are annotated in the reference genome we used here (see Dryad submission associated with Comeault et al. (2020) for genome and annotations). 484 of these genes are found on autosomes and 174 on the *X* chromosome. We have provided tables that describe the genomic locations of each of these gene annotations, gene IDs and names, and the comparison(s) in which they were identified as outliers (Supporting Information Data). This gene set contains genes with interesting functional annotations that include the breakdown of chemicals (e.g., cytochrome P450s; Feyereisen, 1999) and detecting environmental stimuli (e.g., odorant receptors; Brand et al., 2018; Hekmat‐Scafe et al., 2002; Matsuo et al., 2007). However, because we took an outlier‐based approach to identify putatively selected regions, differentiation due to demographic events experienced by populations across *Z*. *indianus's* range could confound our ability to identify truly selected regions of the genome. Some outlier regions support this hypothesis, where almost the entire scaffold shows elevated differentiation (*F*_ST_; see Figures S7–S13). This pattern makes it difficult to identify specific targets of selection. Other regions display localized differentiation within a scaffold (Figures S7–S13). If differentiation was solely driven by demography, we would not expect the latter pattern of localized genetic differentiation. That said, it is important for future work to disentangle the effects of demography, selection, and genomic features, such as local recombination rates, in generating observed patterns of differentiation (Booker et al., 2020; Li et al., 2012). Combining genomic and phenotypic data with experiments estimating fitness in different environments would help to address this challenge. For example, functional analyses of candidate genes could be used to test whether genetic differentiation has resulted in associated shifts in phenotypic traits of interest (Brand et al., 2020). One could then use experiments or estimates of selection in natural populations to confirm the role of particular genotypes and/or phenotypes in affecting an individuals’ performance or fitness in different environments (Barrett et al., 2008; Marques et al., 2017; Nosil et al., 2018; Powell et al., 2020).

### 4.4 The source of putatively adaptive genetic variation in introductions

We found that regions of the genome that are strongly differentiated between introduced and native populations of *Z*. *indianus* harbour both west‐ and east‐African ancestry, suggesting that admixture between these two lineages has played a role in introducing adaptive genetic variation into the population expanding across the western hemisphere. The relative frequency of genomic windows with shared east or west‐African ancestry is different between differentiated windows and the genomic background (compare Figures 5b and 3b, respectively). In order to better understand the adaptive consequences of admixture in populations of *Z*. *indianus* further work that identifies potential traits affecting fitness, and their underlying genes, is needed. Quantitative‐trait locus and admixture mapping represent promising approaches through which this goal could be achieved (Buerkle & Lexer, 2008; Malek et al., 2012; Powell et al., 2020; Rieseberg et al., 2003). Without knowledge on the specific phenotypes contributing to fitness in different environments across *Z*. *indianus's* introduced and native ranges we cannot comment on the relative importance of admixture for adaptation in this system. However, highly differentiated genomic windows that harbor east‐ or west‐African ancestry provide an obvious starting point from which to explore links between genetic variation, phenotypic variation, and fitness. Identifying these links could provide valuable insight into the geographic and evolutionary origins of the genetic variation that help facilitate successful range expansions.

## 5 CONCLUSIONS

Understanding when and where admixture and local adaptation occur during the evolutionary history of introduced species is an important challenge in modern evolutionary biology. Previous work has shown how introduced species can experience admixture within their introduced range and adapt to novel environments they experience (Barker et al., 2017; Colautti & Barrett, 2013; Dlugosch & Parker, 2008; Gibson et al., 2020; Kolbe et al., 2004; Olazcuaga et al., 2020; Popovic et al., 2020; Simon et al., 2020). However, adaptations that help facilitate range expansions associated with human activities may also evolve in the invading species prior to colonization and range expansion (Hufbauer et al., 2012). Here we have provided support for the latter scenario in populations of *Z*. *indianus*, where there is a signature of admixture and selection that is shared across geographically widespread populations in the western hemisphere. Further work is needed to increase our understanding of the adaptive consequences of admixture and the timing of selection on phenotypes that help to facilitate range expansions displayed by many invasive, weedy, or human‐commensal species.

## CONFLICT OF INTEREST

The authors declare no conflicts of interest.

## AUTHOR CONTRIBUTIONS

Aaron A. Comeault and Daniel R. Matute designed the study and conducted fieldwork; Aaron A. Comeault collected genomic data; Aaron A. Comeault and Andreas F. Kautt conducted population‐genomic analyses; Aaron A. Comeault wrote the manuscript with input from all authors. All authors approved the final manuscript and declare no conflicts of interest.

## Supporting information

Supplementary Material

## Data Availability

Illumina sequence data: NCBI short‐read archive: BioProject PRJNA604690. Reference genome and annotations: Dryad: https://doi.org/10.5061/dryad.866t1g1n3
Supplementary data tables: Dryad: https://doi.org/10.5061/dryad.2jm63xspv
Analyses pipelines and scripts: Zenodo: https://doi.org/10.5281/zenodo.4918294
Supporting information available as online document.
