# Supplementary material for: Genomic signatures of admixture and selection are shared among populations of Zaprionus indianus across the western hemisphere
Source: Mol Ecol. 2021 Jul 21;30(23):6193–210. doi: 10.1111/mec.16066 (PMC9290797; doi:10.1111/mec.16066)
Supplement: Supplementary file 1 — Supplementary Material [file MEC-30-6193-s001.docx]

**Supplemental Information for:**

**Genomic signatures of admixture and selection are shared among populations of *Zaprionus indianus* across the western hemisphere**

Aaron A. Comeault*, Andreas F. Kautt, and Daniel R. Matute

*Correspondence: [a.comeault@bangor.ac.uk](mailto:a.comeault@bangor.ac.uk)

**Table of Contents:**

| **Supplementary Methods** | Page 2-7 |
| --- | --- |
| **Supplementary References** | Page 8 |
| **Supplementary Tables** | Page 9-12 |
| **Supplementary Figures** | Page 13-27 |

**Supplementary Methods**

*Sequencing*

For genomes sequenced as part of this study, we first extracted DNA from each individual using a Gentra Puregene Tissue Kit (Qiagen, Valencia, CA, USA) following the recommended tissue protocol with volumes of reagents as suggested for processing 5 - 10 mg of tissue. Genomic DNA libraries were prepared for each individual using KAPA HyperPrep kits (Roche Sequencing, Pleasanton, CA) with a target fragment size of 300-500 bp at the University of North Carolina (UNC) School of Medicine’s high-throughput sequencing facility. Individually barcoded libraries were pooled into groups of 10 to 16 individuals and each pool was sequenced on a single lane of an Illumina HiSeq 4000 machine, generating paired-end 150 bp reads. This sequencing strategy yielded between 4,900 and 14,000 Mb of raw sequence data for each individual (~20 to 50x sequencing depth after mapping and filtering).

*Identifying scaffolds likely belonging to the* X *chromosome*

One challenge with our assignment method was to define a sensible normalized coverage threshold, since we had no prior information on the relative size of the X chromosome in *Z. indianus*. For example, if half of the genome was located on the X chromosome, we would expect each half of the genome to be represented by scaffolds with a mean normalized coverage of either around 0.67 or 1.33, providing a good expectation for the distribution and threshold to use. In our case, with a likely smaller but unknown proportion of the genome located on the X chromosome, defining a threshold is less straightforward. To partly account for this issue, we tried different thresholds ranging from 0.5 to 0.95 in steps of 0.05. For each of these thresholds, we implemented an iterative approach consisting of five rounds in which we first i) classified scaffolds based on a given threshold and then ii) removed previously classified X-scaffolds before re-normalizing coverage for the remaining scaffolds (based on an updated mean overall coverage without X-scaffolds) before repeating step i). We decided on a threshold of 0.85, because the proportion of scaffolds classified as belonging to the X-chromosome seemed to converge around this threshold (Supplementary Data). We acknowledge that using a different threshold might slightly change the number of scaffolds assigned to the X, but we believe it unlikely to affect any of our general conclusions.

*Phasing data using Shapeit*

Before running Twisst we phased our genome-wide SNP dataset using read-aware phasing (Shapeit v2.837; (Delaneau, Howie, Cox, Zagury, & Marchini, 2013)). We extracted haplotypes generated by the *-assemble* tool in Shapeit for each individual in genomic windows consisting of 500 SNPs along the 40 largest scaffolds of the reference genome used for this study (61.2 Mb of sequence, ~42% of the genome). Aligned haplotypes were converted to phylip format and we estimated maximum likelihood trees for each 500-SNP alignment with optimization of substitution rates under the GTR + GAMMA substitution model and 20 runs on distinct starting trees. In addition to the trees we built across the 40 largest scaffolds, we phased genotypes, generated alignments, and constructed trees in 500 SNP windows along any scaffold that contained an outlier window in our analysis of the population branch statistic. This generated a total of ~16,124 gene trees (note that the total number of trees differed among ‘focal’ population datasets, as some windows did not contain polymorphisms for certain population). The number of trees built across each scaffold ranged from 27 (scaffold 227) to 899 (scaffold 2) and the size of the genomic window spanned by each 500 SNP window (from the first SNP to the last SNP) ranged from 1,033 bp to 26,492 bp (median = 4,116 bp; 5% empirical quantile = 2,721 bp, 95% empirical quantile = 6,616 bp).

*Estimating gene-trees using alternate approach*

In the main text we report results from Twisst when run on gene trees constructed using RAxML (maximum likelihood; ML) and the GTR+GAMMA substitution model. This may not be the most appropriate approach for SNP data. We confirmed that our results were not affected by constructing gene trees using RAxML by running Twisst on a set of neighbors-joining (NJ) gene trees that were built from pairwise distance matrices estimated from alignments containing 500 SNPs each (*dist.gene(method=”pairwise”)* and *nj()* functions in R; analysis conducted on 5,870 alignments across the 10 largest scaffolds of the assembly). Topology weights estimated using ML or NJ gene trees were highly correlated across genomic windows (all *p* < 2.2e-16; *r* > 0.51), the proportion of windows that support each possible topology (+ambiguous support) did not differ when using ML or NJ trees (χ^2^ tests; all *p*=0.21), the proportion of windows that were assigned to the same topology when using ML and NJ trees was higher than expected by chance (χ^2^ = 2411; *p*<2.2e-16; 54.1 to 61% of windows were classified the same across approaches, depending on the focal population being analyzed), and the correlation in ancestry between introduced populations in the western hemisphere (east USA and Colombia populations compared: rho = 0.61; *p* < 2.2e-16) was stronger than the correlation between populations in the western hemisphere and India (e.g., USA and India populations: rho = 0.37; *p* < 2.2e-16). We also note that our alignments contain 9 *Z. africanus* individuals. As a result, there are a larger number of sites with between-species substitutions from which the model can estimate substitution rates (see Figure S5 for the number of sites where there is variation segregating among *Z. indianus* samples in our alignments, out of the 500 SNPs in the alignments). Therefore, using the GTR+GAMMA model may be more appropriate than for a ‘within-species-only’ SNP dataset. For reasons listed above we are confident that our results are robust across different approaches used to generate the gene trees we analyzed with Twisst.

*Demographic modeling*

To explicitly estimate the magnitude of change in population size for North American and African populations of *Z. indianus*, as well as the time of divergence, we estimated demographic parameters from the joint site frequency spectrum (jSFS) fastimcoal2 v.2.6.0.3 (Excoffier, Dupanloup, Huerta-Sánchez, Sousa, & Foll, 2013). We fit demographic models using the jSFS derived from the eastern USA population in the western hemisphere and the populations in São Tomé and Zambia in Africa. We chose the population from the eastern USA because we had the largest sample size from this location (N=29) and the two African populations because they were consistently differentiated across analysis (see Figs 1 & 4 in the main text). We polarized the jSFS by assuming that the most common allele observed across the 7 *Z. africanus* individuals in our dataset was the ancestral allele.

We then compared a number of demographic models by fitting simulated SFS to the empirical unfolded jSFS. For each model, we ran 100 independent fastsimcoal runs starting from different initial parameter values. In each run the simulated SFS was estimated with 200,000 coalescent simulations and parameters were optimized during 100 ECM cycles (--maxlhood --dsfs --multiSFS -C 1 --numloops 100 --numsims 200000 --logprecision 18 --brentol 0.0001). The reported log_10_-likelihoods were converted to ln-likelihoods prior to calculation of Akaike Information Criterion (AIC) scores.

*Genome scan with BayPass*

In addition to the PBS analysis reported in the main text we used BayPass (v2.2; (Gautier, 2015)) to identify SNPs with accentuated differentiation between the samples collected in the Western Hemisphere and those from Africa and test for evidence of shared selection across all introduced populations. BayPass implements a Bayesian hierarchical model (Coop, Witonsky, Di Rienzo, & Pritchard, 2010; Gautier, 2015) to estimate loci that show accentuated differentiation among populations and allows the user to specify population specific covariables to test for associations between loci and those covariables. When identifying loci associated with the user-defined covariables, BayPass also estimates a population covariance matrix from the allele frequency data and uses this covariance matrix to account for correlated allele frequencies that are the result of shared population histories. We ran a covariate model in BayPass that included invasion status as the covariate of interest (introduced populations: east USA, Hawaii, and Colombia; native populations: São Tomé, Senegal, Kenya, and Zambia). SNPs that showed allele frequency differences between introduced and native populations were identified using the C2 contrast statistic (Olazcuaga et al., 2020). Because p-values across SNPs were poorly behaved (i.e., the distribution of p-values was multimodal), we followed the recommendation of the authors of BayPass (v2.2; see user manual) and estimated empirical p-values using a pseudo-observed dataset approach. We first simulated allele frequencies at 50,000 SNPs under our observed covariance matrix using the simulate.baypass() R function (Gautier, 2015). We then ran BayPass using these simulated allele frequencies in the same way as for our empirical data. Finally, we estimated empirical p-values and false discovery rates (FDRs) by calibrating our empirical *C*_2_ statistics with estimates from the 50,000 simulated (neutral) allele frequencies. To generate empirical p-values and FDRs we used the empPvals() and qvalue() R functions, respectively (qvalue package; (Storey, Bass, Dabney, & Robinson, 2017)). We considered SNPs with an empirical p-value < 0.05 and FDR < 0.1 as the best candidates for being subject to selection between *Z. indianus*’s introduced and native ranges.

**References**:

Coop, G., Witonsky, D., Di Rienzo, A., & Pritchard, J. K. (2010). Using environmental correlations to identify loci underlying local adaptation. *Genetics*, 185(4), 1411–1423. doi: 10.1534/genetics.110.114819

Delaneau, O., Howie, B., Cox, A. J., Zagury, J.-F., & Marchini, J. (2013). Haplotype Estimation Using Sequencing Reads. *The American Journal of Human Genetics*, *93*(4), 687–696. doi: 10.1016/j.ajhg.2013.09.002

Excoffier, L., Dupanloup, I., Huerta-Sánchez, E., Sousa, V. C., & Foll, M. (2013). Robust Demographic Inference from Genomic and SNP Data. *PLOS Genetics*, *9*(10), e1003905. doi: 10.1371/journal.pgen.1003905

Gautier, M. (2015). Genome-Wide Scan for Adaptive Divergence and Association with Population-Specific Covariates. *Genetics*, 201(4), 1555–1579. doi: 10.1534/genetics.115.181453

Olazcuaga, L., Loiseau, A., Parrinello, H., Paris, M., Fraimout, A., Guedot, C., … Gautier, M. (2020). A Whole-Genome Scan for Association with Invasion Success in the Fruit Fly *Drosophila suzukii* Using Contrasts of Allele Frequencies Corrected for Population Structure. *Molecular Biology and Evolution*. doi: 10.1093/molbev/msaa098

Rabiee, M., Sayyari, E., & Mirarab, S. (2019). Multi-allele species reconstruction using ASTRAL. *Molecular Phylogenetics and Evolution*, *130*, 286–296. doi: 10.1016/j.ympev.2018.10.033

Storey, J. D., Bass, A. J., Dabney, A., & Robinson, D. (2017). qvalue: Q-value estimation for false discovery rate control. R Package Version 2.15.0.

**Supplementary Tables**

**Table S1.** Number of individuals (or samples for the genome from Punjab, India, which was sequenced from two individuals from the same isofemale line) and sample locations included in this study.

| species | Location* | Years | *N* |  |
| --- | --- | --- | --- | --- |
| *Z. indianus* | Florida, USA | 2016 | 2 | This study |
| *Z. indianus* | North Carolina, USA | 2016, 2017, 2018 | 13 | Comeault et al. 2020 + this study (9) |
| *Z. indianus* | New Jersey, USA | 2016 | 2 | This study |
| *Z. indianus* | New York, USA | 2017 | 2 | This study |
| *Z. indianus* | Pennsylvania, USA | 2018 | 2 | This study |
| *Z. indianus* | Tennessee, USA | 2017 | 4 | Comeault et al. 2020 |
| *Z. indianus* | Hawaii, USA | 2017 | 4 | Comeault et al. 2020 |
| *Z. indianus* | Medellín, Colombia | 2018 | 4 | This study |
| *Z. indianus* | Zambia | 2015 | 6 | Comeault et al. 2020 |
| *Z. indianus* | Kenya | 2018 | 7 | Comeault et al. 2020 |
| *Z. indianus* | Senegal | 2018 | 14 | Comeault et al. 2020 |
| *Z. indianus* | São Tomé | 2018 | 6 | Comeault et al. 2020 |
| *Z. indianus* | Punjab, India |  | 1 | Khanna and Mohanty 2017 |
| *Z. gabonicus* | Gabon^!^ |  | 1 | This study |
| *Z. africanus* | Kenya | 2018 | 5 | Comeault et al. 2020 |
| *Z. africanus* | São Tomé | 2018 | 4 | Comeault et al. 2020 |
| Total |  |  | 77 |  |

*Additional details of individual samples and sample locations are given in “supp_data_table0_sample_details.txt”, available in the Dryad repository associated with this manuscript.

^!^This individual was from an isofemale line presumably collected in Gabon, but details are not known.

**Table S2.** Median (pairwise) genome-wide *F*_ST_ estimated across non overlapping 5kb genomic windows.

| ***F*_ST_** | **pop1** | **pop2** | **comparison type** |
| --- | --- | --- | --- |
| 0.160 | Colombia | Hawaii | within introduced range |
| 0.042 | Colombia | East USA | within introduced range |
| 0.089 | Hawaii | East USA | within introduced range |
| 0.166 | Colombia | Zambia | between introduced & native |
| 0.155 | Colombia | São Tomé | between introduced & native |
| 0.230 | Hawaii | Zambia | between introduced & native |
| 0.220 | Hawaii | São Tomé | between introduced & native |
| 0.154 | East USA | Zambia | between introduced & native |
| 0.155 | East USA | São Tomé | between introduced & native |
| 0.077 | Zambia | São Tomé | within native range |
| 0.066 | Kenya | Zambia | within native range |
| 0.084 | Kenya | São Tomé | within native range |
| 0.044 | Senegal | Zambia | within native range |
| 0.054 | Senegal | São Tomé | within native range |
| 0.050 | Kenya | Senegal | within native range |

**Table S3**. Scoring of the best-fit model runs under each of 24 different demographic scenarios fit using Fastsimcoal2. Scenarios are ordered based on increasing AIC score. The best-supported histories are therefore those at the top of the table. MaxEstLhood=estimated maximum log likelihood. The maximum observed log likelihood (MaxObsLhood) given the empirical SFS (i.e. the maximum possible) was -31897403.442.

| **demographic scenario** | **MaxEstLhood** | **N_param** | **AIC** |
| --- | --- | --- | --- |
| F_allgrowth | -32077487 | 13 | 1.47722E+08 |
| G_allgrowth | -32079140 | 13 | 1.47730E+08 |
| B_allgrowth | -32085787 | 12 | 1.47761E+08 |
| D_allgrowth | -32099774 | 11 | 1.47825E+08 |
| F_change | -32103120 | 16 | 1.47840E+08 |
| E_allgrowth | -32105796 | 13 | 1.47853E+08 |
| F_constant | -32112092 | 10 | 1.47882E+08 |
| H_allgrowth | -32113656 | 13 | 1.47889E+08 |
| C_allgrowth | -32132773 | 11 | 1.47977E+08 |
| E_change | -32187536 | 16 | 1.48229E+08 |
| B_change | -32212341 | 15 | 1.48343E+08 |
| G_change | -32216706 | 16 | 1.48363E+08 |
| E_change | -32221548 | 16 | 1.48386E+08 |
| H_change | -32222377 | 16 | 1.48390E+08 |
| C_change | -32294407 | 14 | 1.48721E+08 |
| D_change | -32325069 | 14 | 1.48862E+08 |
| H_constant | -32470232 | 10 | 1.49531E+08 |
| G_constant | -32472894 | 10 | 1.49543E+08 |
| B_constant | -32475026 | 9 | 1.49553E+08 |
| D_constant | -32484747 | 8 | 1.49598E+08 |
| E_constant | -32512556 | 10 | 1.49726E+08 |
| C_constant | -32528613 | 8 | 1.49800E+08 |
| A_change | -32562778 | 14 | 1.49957E+08 |
| A_allgrowth | -32689301 | 11 | 1.50540E+08 |
| A_constant | -32692062 | 8 | 1.50553E+08 |

**Table S4**. Parameter estimates from the highest scoring run (lowest AIC score) under our best-fit demographic scenario (scenario F; Figure S2 below). Column names with N are population sizes, for each lineage (“anc” = ancestral African; “0” = São Tomé; “1” = Zambia; “2” = east USA), in the past (“_0”) and the present (“_1”). Migration rates between the two African lineages are given in columns “M01” and “M10”. The timing of divergence (T1 & T3) and admixture (T2) are reported in units of generations × 1,000 and population sizes as the number of individuals × 1,000.

| **Nanc** | **N0_0** | **N0_1** | **N1_0** | **N1_1** | **N2_0** | **N2_1** | **M01** | **M10** | **ADMIX** | **T3** | **T2** | **T1** |
| --- | --- | --- | --- | --- | --- | --- | --- | --- | --- | --- | --- | --- |
| 5,032 | 52 | 13,190 | 6,942 | 8,963 | 0.15 | 82 | 2.68^-08^ | 4.27^-09^ | 0.9977 | 0.603 | 428 | 4,490 |

**Supplementary Figures**

**
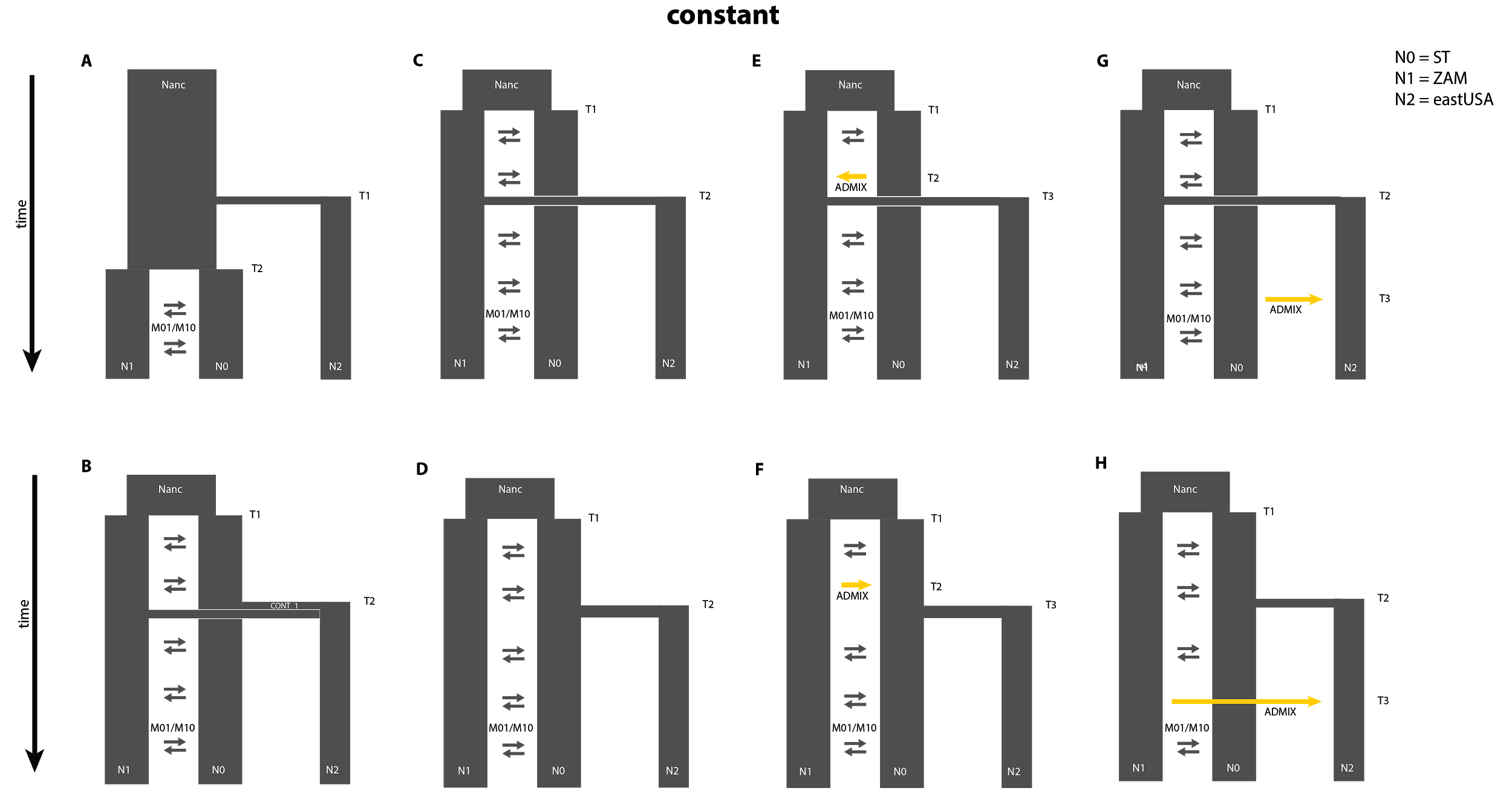
**

**Figure S1.** Demographic models fit to the joint site-frequency spectrum inferred for populations in the eastern USA (N2 = eastUSA), São Tomé (N0 = ST), and Zambia (N1 = ZAM). Population size was held constant within each population for this set of models.

**
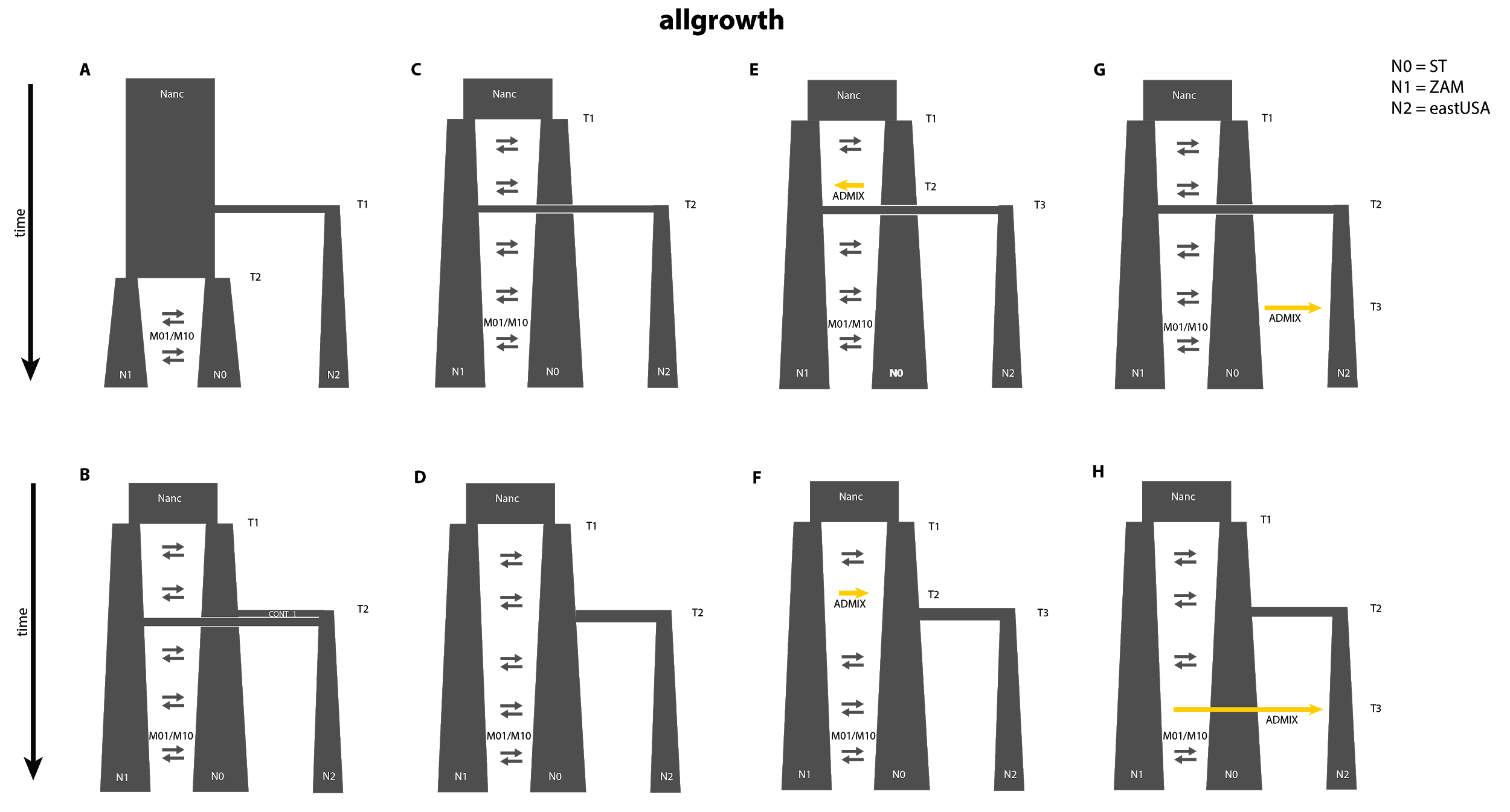
**

**Figure S2.** Demographic models fit to the joint site-frequency spectrum inferred for populations in the eastern USA (N2 = eastUSA), São Tomé (N0 = ST), and Zambia (N1 = ZAM), allowing for exponential growth in each of the extant populations (i.e. N0, N1, and N2). Other than the growth in population size, each demographic scenario is the same as in Figure S1.

**
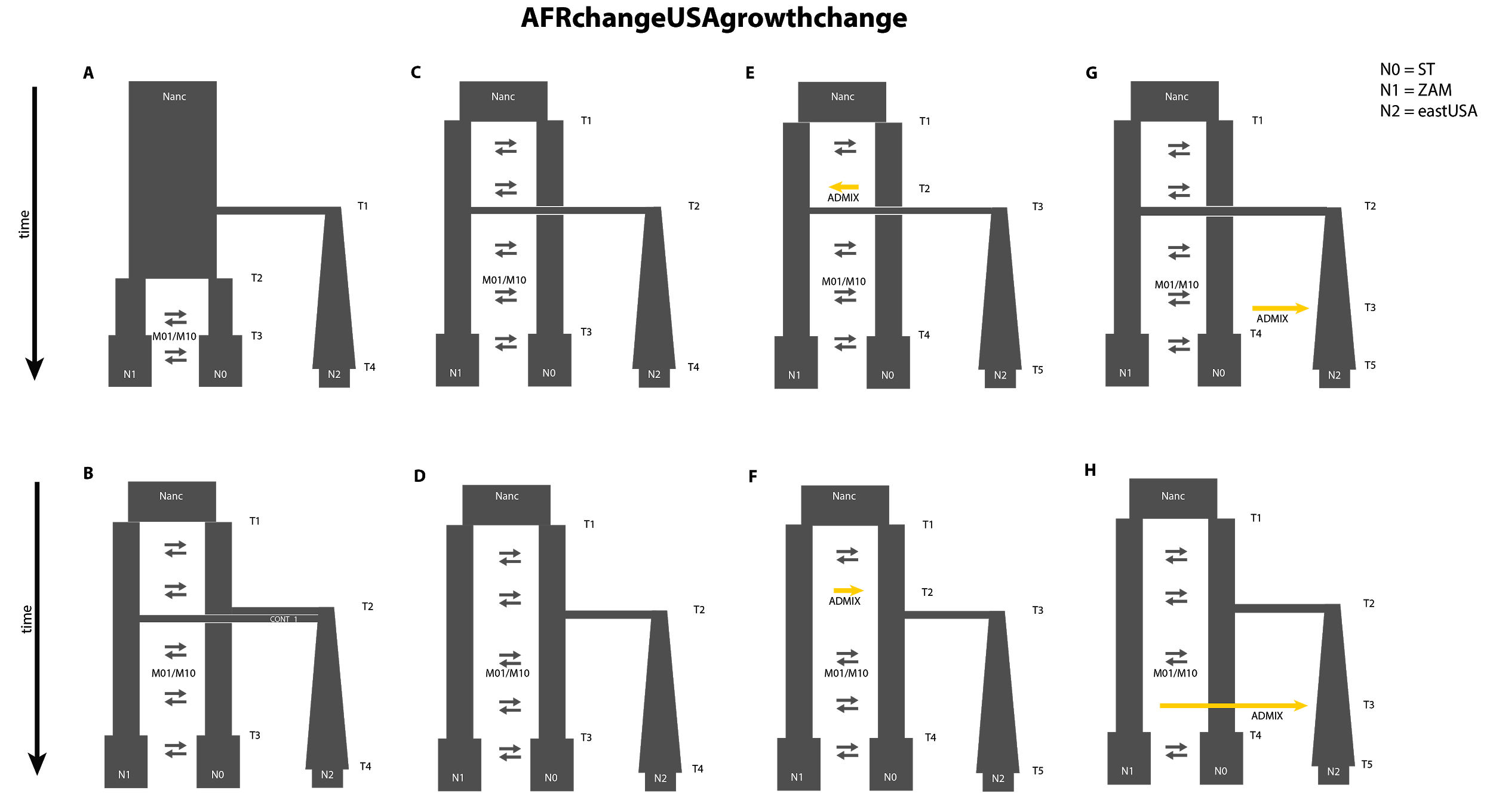
**

**Figure S3.** Demographic models fit to the joint site-frequency spectrum inferred for populations in the eastern USA (N2 = eastUSA), São Tomé (N0 = ST), and Zambia (N1 = ZAM) with exponential growth in the population in the eastern USA and discrete population size changes in each extant population (i.e. N0, N1, and N2). Other than the changes in population size, each demographic scenario is the same as illustrated in Figure S1.

**Figure S4.** Population tree generated using ASTRAL v. 5.6.1 (Rabiee, Sayyari, & Mirarab, 2019). Branch lengths are in coalescent units and quartet support values are reported on internal branches. ASTRAL was run on 7,085 gene trees with options “-t 1” and “-a” specifying individuals belonging to each of the sampling locations indicated in the above tree (tips). Gene trees were generated with RAxML on 500-SNP genomic windows along the 40 largest scaffolds of the genome assembly we used for variant calling (see section “2.3 | Fine-scale relationships across the genome” of the main text for details). The resultant gene trees were thinned by randomly sampling 50% of trees across each scaffold prior to running ASTRAL.


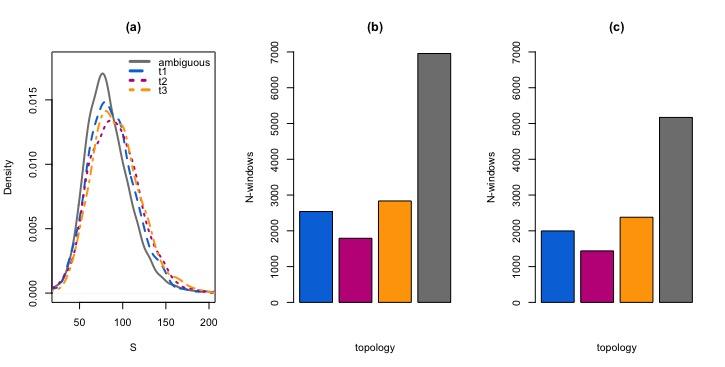


**Figure S5**. The number of segregating sites across *Z. indianus* samples in the alignments used to generate the trees that were then used to estimate topology weights in Twisst. Density plots of the number of segregating sites (*S*) are reported for windows that were classified as ambiguous or supporting topologies 1, 2, or 3 (see main text for topologies). Panels (b) and (c) present the number of windows classified under each topology for all windows (b) and after removing windows where *S* was in the lower 20% across all windows (i.e., *S* > 65; b).


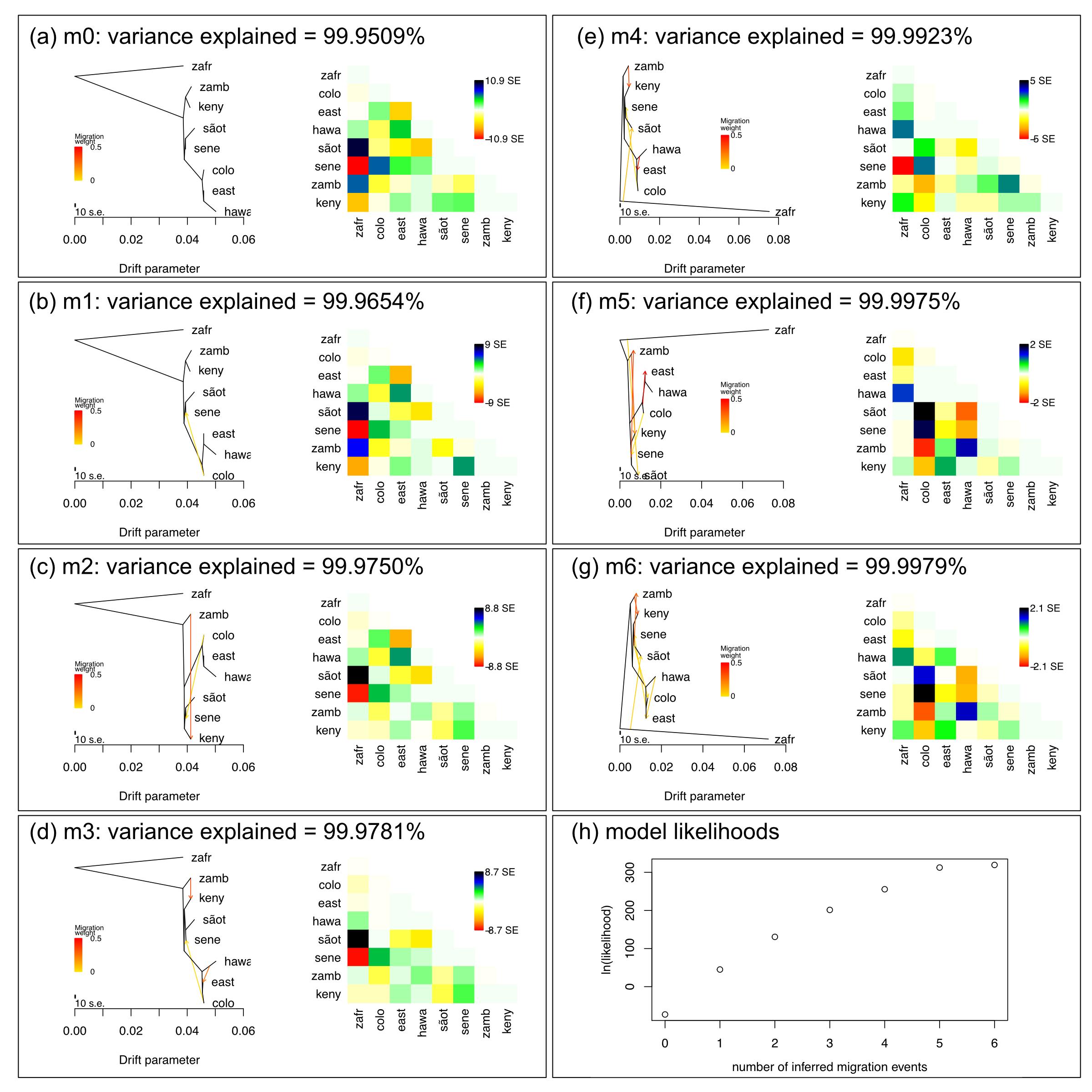


**Figure S6**. (a-g) Admixture graphs inferred using Treemix when specifying the number of admixture events, from 0 to 6, respectively. (h) Log likelihoods for each model specifying the number of migration events ranging from 0 to 6.

**Figure S7**. Genetic differentiation (*F*_ST_) along the five largest autosomal scaffolds that contain at least one outlier window (based on PBS in top 99%). The color of each line represents the introduced population being compared to either the Zambian population (solid lines) or the São Toméan population (dotted lines). Solid grey line is the comparison between the Zambian and São Toméan populations. Genomic windows that are outliers based on PBS in the top 99% of windows in at least two of the introduced populations are indicated with grey boxes.

**Figure S8**. Genetic differentiation (*F*_ST_) along five additional autosomal scaffolds that contain at least one PBS outlier window. Colors are as described for Figure S7 above.

**Figure S9**. Genetic differentiation (*F*_ST_) along five additional autosomal scaffolds that contain at least one PBS outlier window. Colors are as described for Figure S7 above.

**Figure S10**. Genetic differentiation (*F*_ST_) along five additional autosomal scaffolds that contain at least one PBS outlier window. Colors are as described for Figure S7 above.

**Figure S11**. Genetic differentiation (*F*_ST_) along three additional autosomal scaffolds that contain at least one PBS outlier window. Colors are as described for Figure S7 above.

**Figure S12**. Genetic differentiation (*F*_ST_) along five X-linked scaffolds that contain at least one PBS outlier window. Details of colors and boxes are as described for Figure S7 above.

**Figure S13**. Genetic differentiation (*F*_ST_) along five X-linked scaffolds that contain at least one PBS outlier window. Details of colors and boxes are as described for Figure S7 above.

**Figure S14**. Distributions of the number of shared PBS outlier windows expected between different populations under naïve randomization. Distributions are based on 100,000 iterations of randomly selecting the appropriate number of genomic windows within a given set. For example, for the “+ Africa” comparison we selected the same number of outlier windows identified in each of the between-range comparisons and the number of outlier windows identified in both within-native range comparisons and then computed the number of overlapping windows. See Figure 5 in the main text for descriptions of the different comparisons being made.

**Figure S15.** Population structure highlighting individual sample locations and year the individual was collected. Panel (a) is the same as in Figure 1b in the main text and panel (b) is the same as Figure 1c in the main text, other than label details.
